# Longitudinal analysis of human humoral responses after vaccination with a live attenuated *V*. *cholerae* vaccine

**DOI:** 10.1371/journal.pntd.0009743

**Published:** 2021-09-03

**Authors:** Oluwaseyi Adekunle, Alexandra Dretler, Robert C. Kauffman, Alice Cho, Nadine Rouphael, Jens Wrammert

**Affiliations:** 1 Division of Infectious Disease, Department of Pediatrics, Emory University, School of Medicine, Atlanta, Georgia, United States of America; 2 The Hope Clinic, Emory Vaccine Center, Division of Infectious Disease, Emory University, School of Medicine, Atlanta, Georgia, United States of America; University of Maryland School of Medicine, Center for Vaccine Development, UNITED STATES

## Abstract

*Vibrio cholerae* is a bacterial pathogen which causes the severe acute diarrheal disease cholera. Given that a symptomatic incident of cholera can lead to long term protection, a thorough understanding of the immune response to this pathogen is needed to identify parameters critical to the generation and durability of immunity. To approach this, we utilized a live attenuated cholera vaccine to model the response to *V*. *cholerae* infection in 12 naïve subjects. We found that this live attenuated vaccine induced durable vibriocidal antibody titers that were maintained at least one year after vaccination. Similar to what we previously reported in infected patients from Bangladesh, we found that vaccination induced plasmablast responses were primarily specific to the two immunodominant antigens lipopolysaccharide (LPS) and cholera toxin (CT). Interestingly, the magnitude of the early plasmablast response at day 7 predicted the serological outcome of vaccination at day 30. However, this correlation was no longer present at later timepoints. The acute responses displayed preferential immunoglobulin isotype usage, with LPS specific cells being largely IgM or IgA producing, while cholera toxin responses were predominantly IgG. Finally, CCR9 was highly expressed on vaccine induced plasmablasts, especially on IgM and IgA producing cells, suggesting a role in migration to the gastrointestinal tract. Collectively, these findings demonstrate that the use of a live attenuated cholera vaccine is an effective tool to examine the primary and long-term immune response following *V*. *cholerae* exposure. Additionally, it provides insight into the phenotype and specificity of the cells which likely return to and mediate immunity at the intestinal mucosa. A thorough understanding of these properties both in peripheral blood and in the intestinal mucosae will inform future vaccine development against both cholera and other mucosal pathogens.

**Trial Registration:**NCT03251495.

## Author summary

Cholera is a severe diarrheal illness which affects millions of people annually. Those most affected reside in developing countries where outbreaks can cause a devastating healthcare crisis. Despite this, much is still unknown regarding the longevity of protective immune responses or how they are generated and maintained. This includes aspects of the mucosal response where immunity is induced, as well as features of the early immune response that could predict long-term immunity. To address these issues, we examined primary humoral immune responses following administration of a live attenuated cholera vaccine to 12 human participants. This study provides insight into the development of humoral immunity and information that may prove valuable for improving cholerae vaccines and treatments.

## Introduction

*Vibrio cholerae* is the bacterial pathogen responsible for causing cholera, a severe diarrheal illness. There are over three million cases of infection annually resulting in over 95,000 deaths [1]. Cholera is endemic in over 50 countries and, as outbreaks in Yemen highlight, is a persistent global plight, especially where sanitation and clean water supplies fail [2]. The outbreak in Yemen alone was responsible for the doubling of the annual cholera cases reported to the WHO from 2018 to 2019 [3]. Intriguingly, individuals who experience a symptomatic episode of cholera can be protected against subsequent exposure for up to 10 years following the disease [4–6]. In contrast, inactivated vaccines have shown more limited durability of immunity [7]. In this context, the mechanism(s) contributing to durable immunity are not well understood. Consequently, the focus of current research efforts is aimed at understanding the immune factors responsible for protection, and to aid in the development of vaccines that can provide durable immunity [7–10].

Humoral responses against *V*. *cholerae* are thought to be responsible for protective immunity. In endemic areas, the antibodies generated in response to exposure primarily target the two immunodominant antigens of the bacteria, lipopolysaccharide (LPS) and cholera toxin (CT) [11,12]. The LPS specific antibodies play an important role in vibriocidal activity, which measures the ability of antibodies to bind and induce complement mediated lysis of the bacteria [13]. Serum vibriocidal responses are currently the best-known correlate of immunity to *V*. *cholerae* infection in humans [14]. However, serum vibriocidal responses do not fully explain cholera protective immunity. Vibriocidal titers wane well before immunity is lost and there is no absolute threshold which ensures sterilizing immunity [15]. The reason for this discrepancy is unclear. One possibility is that the concentration of antibodies in the serum may not be reflective of the concentration in mucosa where protection is mediated. Moreover, the role of complement mediated lysis as the primary mechanism of immunity within the gut has not been clearly established [16–18]. While the precise mechanism remain unclear, antibodies targeting the o specific polysaccharide of the LPS molecule are associated with protection [8].

*V*. *cholerae* is a non-invasive pathogen and is thus less susceptible to the neutralizing effects of peripherally circulating antibodies. Therefore, protection is likely mediated by antibodies which are secreted into the intestinal lumen by tissue resident plasma cells [19]. Due to the difficulty in examining these tissues in humans, the properties of cholera specific plasma cells at this location are not well understood [20]. Plasmablasts are antibody secreting cells (ASC) that are present transiently in circulation shortly after infection or vaccination [21–23]. Following circulation these cells may die or migrate to sites such as the bone marrow and mucosal tissues where they transition into long lived plasma cells. Therefore, plasmablasts are a readily accessible population of antigen specific cells that can provide insight into the immunological processes that occur in the mucosa and the properties of cells that may ultimately reside in these tissues.

CVD 103-HgR, currently sold under the trade name Vaxchora, is a live attenuated *V*. *cholerae* strain that is approved in the United States as a traveler’s vaccine [9,24]. It is modified from the classical Inaba 569B strain [25,26]. While the primary attenuation is the deletion of 94% of the A_1_ toxin subunit, other modifications include the absence of the El tor hemolysin, a shiga like toxin, and the insertion of a mercury resistance marker. The vaccine has proven to be very effective in short term studies, with 80% of human volunteers protected from disease following challenge three months post vaccination [27], and the remaining 20% showing significantly reduced shedding. The availability of this vaccine provides the opportunity to safely examine the humoral response to an intestinal pathogen in a naïve cohort [26,28,29]. Understanding the induction of plasmablasts, memory B cells, antibody isotype, gut homing potential, and antigen binding in a naïve host and determining of how these factors change longitudinally is important not only for our understanding of *V*. *cholerae* immunity but can also serve as a model to better understand mucosal immunity. Additionally, the longitudinal aspect of this study provides the opportunity to identify which characteristics of the early immune response are predictive of durable immunity. This information will be helpful in focusing future vaccine improvements. Finally, comparing these responses to natural infection will provide important information regarding key differences between primary immune responses and recall responses in highly endemic areas. In accordance with these goals, we conducted a longitudinal study to examine how components of both the cellular and serological humoral response change in vaccinated individuals and evaluated markers that may reflect the generation and maintenance of mucosal immunity.

## Methods

### Ethics statement

This study was approved by the institutional review board of Emory University. Informed written consent was obtained from all participants prior to participation within the study.

### Study design

12 study participants were enrolled from the metro Atlanta area in Georgia. Demographics were 5 female, 6 male, and 1 other with an age range of 22–49 years. 8 participants identified as white, 3 identified as Black or African American, and 1 identified as both white and American Indian or Alaska Native. 1 participant was Hispanic, the remaining 9 were non-Hispanic. Participants were in good health as determined by medical history and physical exam and had no history of prior cholera vaccination or disease. Written informed consent was obtained from subjects prior to any study procedure. Subjects were given a description of the study prior to enrollment. Exclusion criteria included any acute or chronic medical condition, medication, or disorder that would make vaccination unsafe, prior history of cholera vaccination or cholera disease. Each subject made a total of 7 visits to Emory’s Hope Clinic over the course of a year during which blood samples were collected. In the first visit blood from each subject was collected prior to administration of a single dose of the oral live cholera vaccine (Vaxchora) according to manufacturer’s instruction. Subjects remained in the clinic for observation for an additional 20 minutes. In addition, subjects fasted at least one hour prior to and one hour after vaccination. Vaccination and blood collections were performed at the Hope Clinic, the clinical arm of the Emory Vaccine Center following good clinical practices. The vaccine was well tolerated and reactogenicity in the first week after vaccination was mostly mild and moderate side effects. Of the mild and moderate side effects, the most common was headache followed by fatigue, diarrhea, abdominal pain, and nausea/vomiting. No subjects experienced any grade 3 or higher adverse events. 1 unsolicited adverse event of ecchymosis following blood draw was noted. No severe adverse reactions occurred. This study was approved by the Emory institutional review board. Further details on this clinical trial are located at clinicaltrials.gov under NCT03251495.

### Blood collection and processing

Blood was collected by venipuncture prior to vaccination, and on days 7, 10, 15, 30, 90 and 365. Approximately 64 mLs of blood were drawn into CPT tubes at each timepoint. Additionally, approximately 3 mLs of blood were drawn into serum tubes. Blood was centrifuged at 1600 rcf for 30 min. The serum fraction was removed and aliquoted for further studies. The cell layer was harvested and lysed with 5 mLs of ACK lysis buffer (Quality biological #118-156-101) for 5 minutes. After which cells were washed with PBS + 2% FBS three times after spinning at 1200 rpm for 8 minutes. Cells were then resuspended in the appropriate buffer for counting and further experiments.

### Flow cytometry and single-cell sorting

Immunophenotyping of the B cell subsets was performed on PBMC after staining with the following antibodies: CD27-APC (O323), CD3-AF700 (HIT3a), CD71-FITC (CY1G4), IgD-PerCPCy5.5 (IA6-2), CCR9-PE (BBC3M4), CD19-PECF594 (HIB19), CD38-PECy7 (HIT2), CD20-V450 (L27), IgA-APC (ISs11-8E10), CD14-AF700 (61D3), CD16-AF700 (CB16), IgG-PECy7 (G18-145), IgM-BV650 (MHM-88), and CD27-BV711 (O323). For each panel, a total of 2 million PBMC were stained. A minimum of 100,000 events were acquired on a BD LSR II Flow Cytometer and data was analyzed using FlowJo software.

### ELISPOT assay

ELISPOT was performed to enumerate cholera toxin and LPS specific plasmablast present in the PBMC samples. 96-well ELISPOT assay filter plates (Millipore) were coated overnight at 4°C with CTB (List Labs #104), LPS (generously provided by Dr. Edward Ryan’s Laboratory), or polyvalent goat anti-human Ig (10 μg/mL, Jackson ImmunoResearch) in PBS. Plates were washed with PBS Tween 0.05% and blocked with R10 (RPMI 1640 + 10% FBS +1% P/S +1% L-glutamine) at 37°C for 2 hours. Freshly isolated PBMC were added to the plates in a dilution series starting at 5 x 10^5^ cells and incubated overnight at 37°C. The following day plates were washed with PBS, followed by PBS Tween 0.05%, and incubated with biotinylated anti-human IgG, IgA, or IgM antibody (Invitrogen) at room temperature for 90 minutes. After washing plates were incubated with avidin D-horseradish peroxidase conjugate (Vector laboratories) and developed using 3-amino-9-ethyl-carbazole substrate (Sigma-Aldrich). Plates were scanned and analyzed suing an automated ELISPOT counter (CTL, Cellular Technologies).

### Memory B cell assay

Antigen-specific memory B cells (MBC) were detected essentially as previously described [30]. Briefly, PBMC were cultured at 1x10^6^ cells per ml of R10 supplemented with 50 uM B-mercaptoethanol (Sigma-Aldrich) and polyclonally stimulated with pokeweed mitogen extract (1 μg/mL, Sigma-Aldrich), phosphothiolated CpG ODN-2006 (6 μg/mL, Invivogen), and Staphylococcus aureus Cowan (1:10,000, Sigma-Aldrich) for 6 days. After *in vitro* stimulation, total and *V*. *cholerae* specific IgM, IgG, and IgA cells were quantified by ELISPOT assay, as described above.

### ELISA assay

MaxiSorp plates were coated with CTB (List labs #104) at 1μg/mL diluted in 50 mM carbonate buffer overnight at 4°C. The following day plates were washed with PBS Tween 0.5% then blocked with PBS 1% BSA for 90 minutes. Plates were washed then incubated with serum diluted in PBS Tween 0.5% 1% BSA for 90 minutes. Plates were washed then incubated with peroxidase conjugated goat anti human IgM (109-036-011), IgG (109-036-098), or IgA (109-036-129) diluted in PBS Tween 0.5% 1% BSA for 90 minutes. Plates were washed with PBS Tween 0.5% 1% BSA followed by PBS. Wells were developed with OPD substrate solution: 0.4 mg/ml of O-phenylenediamine (Sigma #P8787) dissolved into 50 mM citrate buffer (Sigma #P4560) with 30% H_2_O_2_. Plates were incubated with OPD substrate solution for 5 minutes. 100 ul of 1M HCl was added to stop the reaction and O.D. was recorded at 490 nm using the Bio-Rad IMark microplate reader. Seroconversion was defined by responses 4-fold higher than prevaccination samples.

### Vibriocidal assay

Vibriocidal titers were detected essentially as previously described[21]. *V*. *cholerae* O1 (strain 19479 El Tor Inaba) was cultured to mid log phase for 2 ½ hours in bovine heart infusion media at 37°C. Bacteria were pelleted at 3,000xg for 10 min and washed twice with PBS. Prior to use bacteria were normalized to an O.D. of 0.3. Guinea pig complement (Sigma S1639), bacteria, and heat inactivated serum (56°C for 30 minutes) were mixed and added to each well for a total volume of 50 ul per well in a flat bottom 96 well plate. Plates were incubated on shaker (50 RPM) at 37°C for one hour. 150 ul of BHI media was added to each well and plates were incubated at 37°C for approximately 3 hours without shaking until the O.D. at 595 nm of untreated control wells was between 0.20–0.28. Plates were read with Bio-Rad IMark microplate reader. Seroconversion is defined as titers that are 4-fold higher than day 0 samples. Half-life estimates of the vibriocidal titers were determined on the average titer of all subjects using the exponential decay function y = a*e^(kt) where y is the ending vibriocidal titer (90 or 365 days), a is the peak vibriocidal titer (30 or 90 days), t is time in days after peak vibriocidal titer, and k is the constant rate. Once k has been determined estimated half-life (T½) was calculated using the equation T(½) = (ln(1/2))/k.

### Agglutination assay

Agglutination titers were detected essentially as previously described [21]. *V*. *cholerae* O1 (strain 19479 El Tor Inaba) was cultured to mid log phase for 2½ hours in bovine heart infusion media at 37°C. Bacteria were pelleted at 3,000 x g for 10 min and washed twice with PBS. Prior to use bacteria were normalized to an O.D. of 0.3. Equal amounts of heat inactivated serum (56°C for 30 minutes) and bacteria were mixed and added to a V-bottom microtiter plate for a total of 50 ul per well. Each plate was sealed with an adhesive film, centrifuged for 10 seconds, then incubated for 20 hours at 4°C. Plates were imaged using a UV imaging system (ChemiDoc, BioRad). Agglutination titers were recorded as the last dilution where bacteria were agglutinated.

### Statistical test

Graphs and statistical tests were performed using GraphPad Prism software version 8.0. One-way or a two-way ANOVA was used to determine statistical significance. Information about the statistics used for each experiment, including sample size, experimental method, and specific statistic test employed, can be found in the relevant results section or figure legend.

## Results

### Rapid and durable serological responses following primary vaccination

For the current study 12 healthy volunteers were recruited from the Atlanta, Georgia metropolitan area who had no prior history of *V*. *cholerae* infection, *V*. *cholerae* vaccination, or travel to a cholera endemic area within the past five years. Peripheral blood mononuclear cells (PBMC) and plasma were collected on days 0, 7, 10, 15, 30, 90, and 365 following vaccinations from whole blood to examine both acute and memory timepoints. The serological analyses were focused on functional assays and binding assays against the immunodominant antigens lipopolysaccharide (LPS) and cholera toxin B subunit (CTB).

**Fig 1:**
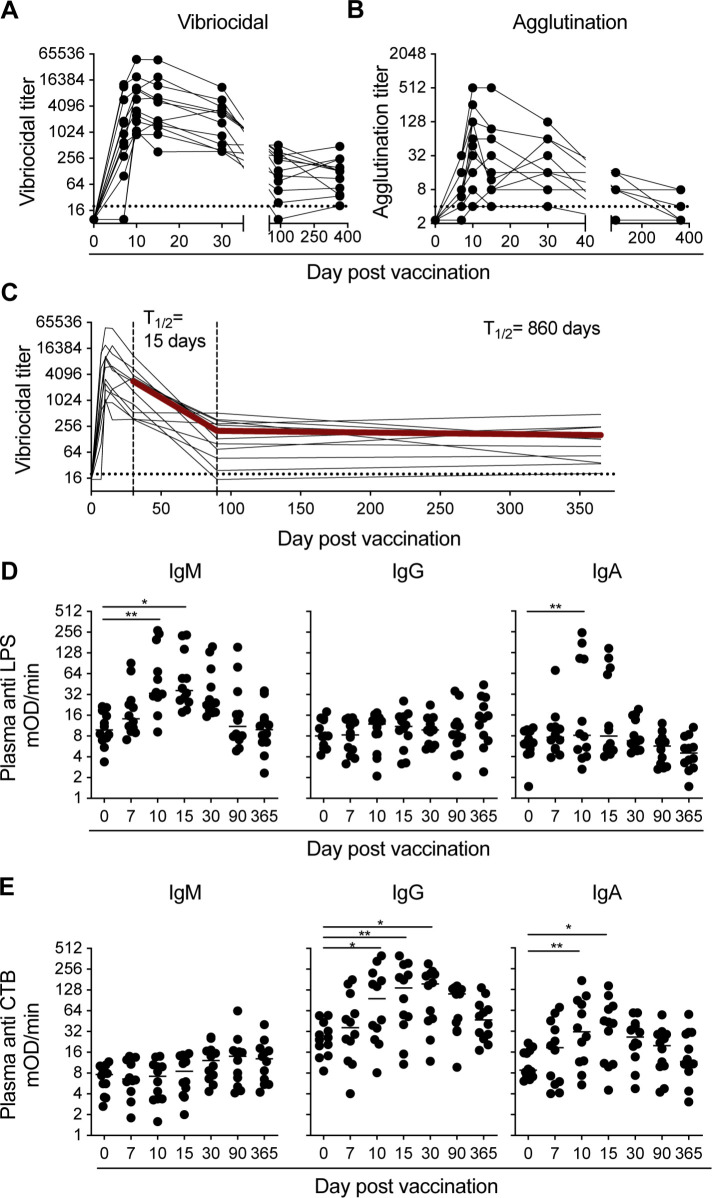
*V*. *cholerae* specific plasma antibody titers rise rapidly following vaccination and are maintained at least for one year post vaccination. (A) Vibriocidal and (B) Agglutination titers on day 0, 7, 10, 15, 30, 90, and 365 for each of the 12 subjects following vaccination. Dotted line in the vibriocidal and agglutination figures indicate the lowest dilution of serum tested in the assay. (C) Longitudinal vibriocidal titers measured for each of the 12 subjects following vaccination from day 0 to day 365. Half-life (T½) estimated by an exponential decay model using the average vibriocidal titers for all subjects (Line bolded in red). Half-life estimate has been calculated from day 30–90 (first set of dashed lines), and from day 90–365 (second set of dashed lines). Dotted line indicates the lowest dilution of serum used in the assay. (C) Anti lipopolysaccharide and (D) anti cholera toxin B subunit titers for IgM, IgG, and IgA plasma antibodies on days 0, 7, 10, 15, 30, 90, and 365 for each of the 12 subjects following vaccination. Statistical significance between bracketed samples was determined using a one-way ANOVA. Significance values are indicated by asterisks (P < 0.05 (*); P < 0.005 (**); P < 0.0005 (***); P <000.1 (****)).

The functional responses focused on the vibriocidal and agglutination titers generated following vaccination. Due to previous reports showing that elevated levels of vibriocidal titers are strongly correlated with lower incidence of infection [27], we examined these responses in our cohort. All vaccinees seroconverted (defined by a four-fold increase from baseline in vibriocidal activity) by 10 days post vaccination **(Fig 1A)**. The vibriocidal titers of the majority of our cohort peaked 10 days post vaccination (GMT 4550, range 902–49400), while a smaller subset of donors peaked on day 15 post vaccination (GMT 3610, range 360–47700). Vibriocidal antibodies were also observed as early as day 7 in 83% of the vaccinees, although at an average of threefold less than the peak of the response. Vibriocidal titers then declined an average of 46-fold from peak levels by day 90 post vaccination. Afterwards, the titers remained stable showing no significant difference between the day 90 and 365 time points (day 90 GMT 125 vs day 365 GMT 110; *p* = 0.378).

Agglutination by antibodies specific for proteins and sugars on the bacterial cell surface is another proposed mechanism of immunity. Vibriocidal assays largely measure IgM and IgG responses due to the ability of these isotypes to activate the classical complement pathway. However, agglutination assays can incorporate IgA responses in the measure of bacterial specific antibodies. This could be particularly relevant if a response generated a strong agglutination titer but a comparably weak vibriocidal titers. In our analysis, the agglutination titers largely mimicked the response kinetics of the vibriocidal titers in which the titers rapidly peaked early in the response. In our cohort, 10 vaccinees reached the peak of the response 10 days after vaccination. Interestingly, while the vibriocidal titers remained relatively stable between days 90 and 365 post vaccination, the agglutination responses continued to decline. Only four of the donors examined had detectable agglutination titers at 1-year post vaccination **(Fig 1B)**. Vibriocidal and agglutination titers correlated positively at every timepoint up to day 30 post vaccination, however, there was not a strong correlation between these two responses after this timepoint.

Vibriocidal titers are currently the best known immune correlate of protection against cholera disease. Therefore, the long-term persistence of these titers following immunization could be indicative of long-term protective potential of the vaccine. The vibriocidal titers in our cohort did not suffer a significant decline in magnitude between day 90 and 365 post vaccination. To estimate the persistence of vibriocidal titers past this time point, we used an exponential decay model to calculate the estimated half-life of the titers in both the acute and memory phase of the immune response (**Fig 1C**). Using this calculation, we estimated the half-life of the vibriocidal titers to be 860 days. Based on this estimation, we predict that these titers will be able to persist at least up to two years post vaccination.

Next, we examined the serum binding responses to the two immunodominant antigens of *V*. *cholerae*, LPS and CTB. A significant increase in LPS specific serum antibody responses, as evidenced by a threefold increase in antibody titers after vaccination, was elicited in 11 out of 12 donors. The LPS specific responses were largely dominated by IgM and IgA antibodies **(Fig 1C)**. These titers followed similar kinetics to the early vibriocidal responses. The IgM and IgA anti LPS titers peaked on days 10 (mean 85 mOD/min, range 9–272) and 15 post vaccination (mean 76 mOD/min, range 17–234) respectively. Eight of the vaccinees (66%) had a three-fold increase over baseline IgM anti LPS titers following vaccination. However, in the majority of these donors, the LPS specific IgM antibody titers returned to levels less than threefold above baseline levels by day 90. In two of the vaccinees, LPS specific antibodies 3-fold above baseline were still present in plasma at day 90; however, by one year, these titers were equivalent to baseline levels. In contrast to the IgM titers, only 4 out of the 12 volunteers had an elevated LPS specific IgA titer following vaccination. The LPS specific IgA titers were more transient than the IgM response, returning to baseline levels in all donors by day 30 post vaccination. In contrast to the vaccine’s ability to induce an IgM and IgA response, its ability to generate LPS specific IgG antibodies was poor in contrast to what is observed following infection [31]. Relative to baseline levels, only one donor had a threefold increase in LPS specific IgG titers following vaccination.

The vaccine strain CVD-103 HgR produces only the cholera toxin B subunit (CTB) as a result of an attenuating deletion of the CTA1 subunit [25,26]. Therefore, we focused our toxin specific serological analyses on responses to CTB **(Fig 1D)**; however, as shown in the **(S1 Fig)**, similar analysis using the cholera holotoxin found no statistical difference in the kinetics or magnitude to either protein. Following vaccination, 75% of our cohort had an increase in plasma antibody titers towards CTB defined as a threefold increase over baseline levels. IgG titers increased above baseline levels in 7 of the volunteers. These responses peaked 15 days post vaccination (mean 155 mOD/min, range 10–400) and remained elevated above baseline in 5 donors 90 days following vaccination (mean 28 mOD/min vs 89 mOD/min; *p* = 0.0025). IgG titers in all but one donor returned to baseline by one-year post vaccination. An analysis of CTB specific IgA serum titers demonstrated that half of the donors had a significant increase following vaccination. These titers peaked on day 10 (mean 168 mOD/min, range 5–174) and returned to baseline 30 days post vaccination in all but one donor, who maintained elevated titers for at least a year post vaccination **(Fig 1D)**. Unlike LPS which generated a robust IgM response, there was no significant increase in CTB specific IgM titers at any timepoint following vaccination. However, it is conceivable that any low affinity CTB specific IgM antibodies that are present may have been outcompeted by the higher affinity IgG and IgA antibodies, as has been described in other systems [32,33].

### Vaccination induces a potent LPS and CTB specific plasmablast response

**Fig 2:**
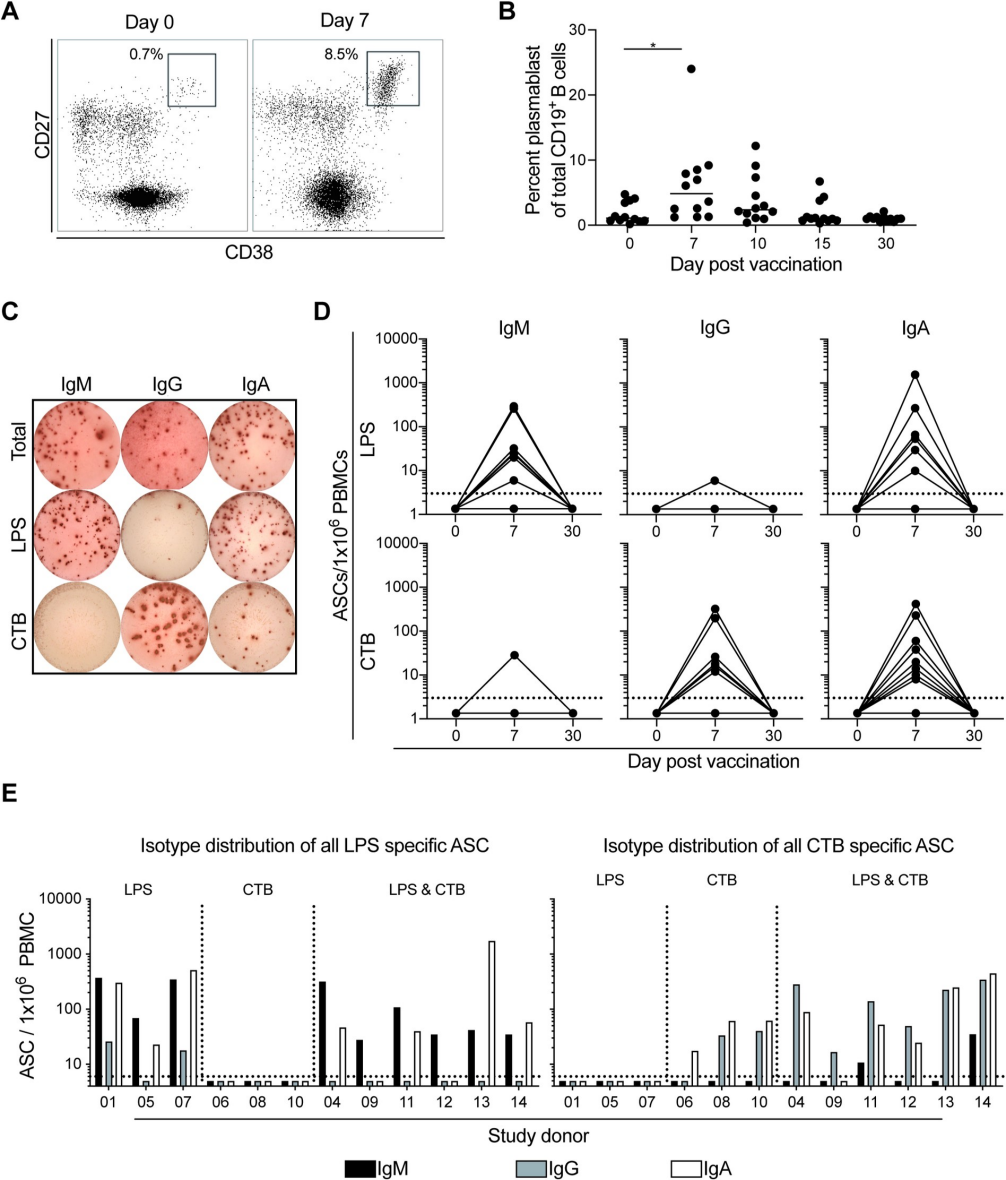
Antigen specific plasmablasts increase following vaccination. (A) Representative flow cytometry plot of the plasmablast population from one donor on day 0 and 7 post vacation. (B) Summary flow cytometry analysis of the plasmablast population following vaccination for each of the 12 participants on day 0, 7, 10, 15, and 30 post vaccination. (C) Representative ELISPOT of the total, LPS, and CTB specific IgM, IgG, and IgA ASC from one subject on day 7 post vaccination. (D) Summary ELISPOT analysis of lipopolysaccharide (LPS) and cholera toxin B subunit specific IgM, IgG, and IgA antibody secreting cells following vaccination from each of the 12 subjects on day 0, 7, and 30 post vaccination. Dotted line indicates limit of detection for the ELISPOT assay. (E) Isotype distribution of IgM (black) IgG (grey), and IgA (white) of all ASC for each of the 12 subjects. Dotted line indicates limit of detection for the ELISPOT assay. Statistical significance between bracketed samples was determined using a one-way ANOVA. Significance values are indicated by asterisks (P < 0.05 (*); P < 0.005 (**); P < 0.0005 (***); P <0.0001 (****)).

Following vaccination there was a significant increase in circulating total plasmablasts (CD3^-^CD19^+^CD27^hi^CD38^hi^ lymphocytes) on day 7 compared to day 0 post vaccination (mean 1.9% vs 6.3%; p = 0.04) **(Fig 2A)**. Seven of the volunteers (58%) showed an increase in the total plasmablast compartment. Of the vaccinees who mounted an increase, five peaked 7 days post vaccination while two peaked 10 days post vaccination **(Fig 2B)**. As expected, given the transient nature of plasmablasts in circulation, their frequency returned to baseline levels in all donors by 30 days post vaccination. This increase in the vaccine induced plasmablast population is consistent to our prior studies examining the plasmablast responses in patients infected with *V*. *cholerae* [21].

To define the kinetics and magnitude of antigen specific plasmablast responses following vaccination we also used the enzyme linked immunospot assay (ELISPOT) **(Fig 2C)**. Nine of the vaccinees had detectable LPS specific IgM ASC following vaccination. Of these nine donors, seven had a peak response at day 7 while two peaked at day 10 **(S2 Fig)**. The peak response had an average of 80 LPS specific IgM ASC/million PBMC (range 0 to 294 ASC/million PBMC). Similar to the LPS specific IgM secreting ASC kinetics, a majority of our study cohort (8 of 12) also had detectable LPS specific IgA ASC. Of these eight donors, five had a peak response at day 7 while three peaked at day 10. In comparison to the LPS specific peak IgM response, the peak IgA response was similar (mean 166 LPS specific IgA ASC/million PBMC; range 0 to 1550). Interestingly, only two donors had detectable LPS specific IgG ASC responses. These two donors also had the highest levels of LPS specific IgM ASC in our cohort. Finally, we determined that the antibodies released by the ASC were also cross reactive with LPS from the Ogawa serotype **(S3 Fig)**.

We next examined the CTB specific ASC responses following vaccination. Similar to the LPS responses, a majority of our cohort mounted CTB specific ASC. However, in contrast to the LPS specific responses, only two donors had detectable CTB specific IgM ASC following vaccination (27 CTB specific IgM/million PBMC). The IgG response was much more robust with 8 donors having detectable CTB specific IgG ASC. The CTB specific IgG ASC also peaked 7 days post vaccination (mean 67 IgG/million PBMC; range 0 to 324) in 6 participants and with an additional 2 vaccines who reached their peak on day 10. Eight of the donors also had a CTB specific IgA ASC response which also peaked on day 7 post vaccination (mean 67 CTB specific IgG ASC/million PBMC; range 0 to 417).

Intriguingly, while in sum all donors responded to the vaccine, we observed that only half of the vaccinees had an ASC response to both the CTB and LPS antigens, while the other half mounted a detectable response directed against only CTB (25%) or only LPS (25%), respectively **(Fig 2E)**. The isotype specificity of the LPS and CTB specific ASCs followed a similar trend to what was seen in the serological analyses. Specifically, the LPS responses were primarily derived from IgM and IgA ASC while the CTB responses were largely derived from IgG and IgA ASC **(Fig 2E)**.

### Vaccine induced plasmablasts express the gut homing receptor CCR9 in an isotype dependent manner

**Fig 3:**
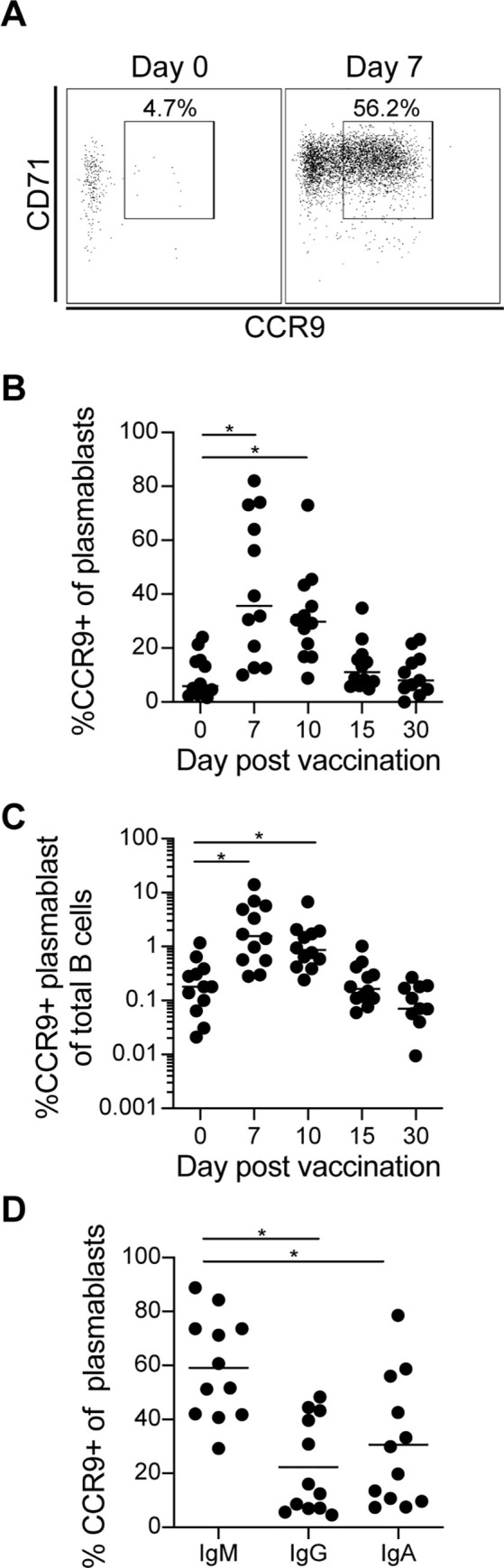
Induction of CCR9 expression on plasmablasts after vaccination. (A) Representative flow cytometry analysis of the percent of CCR9+ cells of the total plasmablasts population on day 0 and day 7 from one study participant. (B) Summary flow cytometry analysis of the percentage of the CCR9+ cells from the plasmablasts population from all 12 subjects on days 0, 7, 15, and 30 post vaccination. (C) Summary flow cytometry analysis of the percent of CCR9+ plasmablasts from the total B cell population from all 12 subjects on days 0, 7, 15, and 30 post vaccination. (D) Percentage of the IgM, IgG, and IgA expressing plasmablasts which were also CCR9+ on day 7 for each of the 12 subjects. Statistical significance between bracketed samples was determined using a one-way ANOVA. Significance values are indicated by asterisks (P < 0.05 (*); P < 0.005 (**); P < 0.0005 (***); P <0.0001 (****)).

As plasmablasts circulate following vaccine exposure, we were interested in the potential of the cells to travel to the mucosa where they may release antibodies into the intestinal tract and/or become long lived plasma cells. CCR9 is a chemokine receptor that binds to C-C motif chemokine ligand 25 (CCL25) secreted by intestinal epithelial cells and helps cells traffic to the small intestine [19,34]. Therefore, using a flow cytometry driven approach we examined the change in CCR9 expression on plasmablasts following vaccination **(Fig 3A)**. This analysis demonstrated a substantial increase in the percentage of CCR9 expressing plasmablasts on day 7 in which an average of 42% percent of the cells expressed the marker, relative to the pre-vaccination expression of 9% **(Fig 3B)**. In some donors, the percentage of CCR9 expressing plasmablasts exceeded 80% (range 8%-82%). As IgA is the principal antibody in mucosal secretions, we also examined CCR9 expression by isotype. On day 7, 59% total IgM+ plasmablasts (range 29% to 89%) had increased expression of CCR9. In contrast, only around 30% of the IgA+ plasmablasts (range 7.4% to 71%) and 22% of the IgG+ plasmablasts (range 4.7% to 48%) plasmablasts expressed CCR9 **(Fig 3D)**.

### Memory B cell responses to vaccination are dominated by IgA isotype

Previous household contact studies have shown a correlation between antigen specific memory and a reduced incidence of infection [35,36]. Therefore, we used an ELISPOT based assay to examine the IgG and IgA MBC responses to LPS and CTB at days 30 and 90 post vaccination. For the LPS specific IgG MBC, only two donors had detectable antigen specific responses following vaccination. Intriguingly, we observed that one of the donors had LPS specific IgG MBC prior to vaccination, however, these cells were not detected on day 30 post vaccination **(S4 Fig)**. Similar to the LPS specific MBC response, only 4 of the volunteers had CTB specific IgG MBC following vaccination. Two of the donors first had detectable responses on day 30, while 4 were detectable on day 90. The relatively low cholera specific IgG MBC, particularly when compared to the robust IgA response, could be indicative of poor MBC generation. Alternatively, it may also indicate that the IgG MBC responses do not circulate systemically but preferentially home and reside in the intestinal or other lymphoid organs. Tissue resident memory B cells are known to occur in humans, however the factors that determine the generation and frequency of these cells following *V*. *cholerae* exposure have yet to be determined [37].

In contrast to the IgG responses, there were a larger number of donors who had detectable IgA MBC for both antigens. By measuring the LPS specific IgA MBC at days 30 and 90, we observed that the vaccine induced an antigen specific MBC response in eleven of the donors. Six of the responders had detectable MBC on both day 30 and 90. The other five donors had responses on day 30 (2), or day 90 (3), respectively. Additionally, two of the donors had LPS specific MBC prior to vaccination and maintained detectable antigen specific MBCs throughout all timepoints tested.

CTB specific IgA MBC were also increased with ten donors displaying specific MBC on both day 30 and day 90 post vaccination. While we detected CTB specific IgA MBC in six donors on both days, the remaining four vaccinees had specific cells only on either day 30 (2) or day 90 (2). The two donors that had detectable CTB specific MBC on day 0 maintained these responses through day 90. Finally, for those individuals that developed an antigen specific MBC response post vaccination, we observed that the magnitude of the LPS and CTB IgA MBC response was similar. Specifically, the LPS specific IgA accounted for approximately 0.2% (range 0 to 0.65) of the total MBC population while the CTB specific IgA MBC accounted for 0.16% (range 0 to 0.72) of the MBC population.

### LPS specific IgM ASC and gut homing IgM plasmablasts correlate with early vibriocidal activity

**Fig 4:**
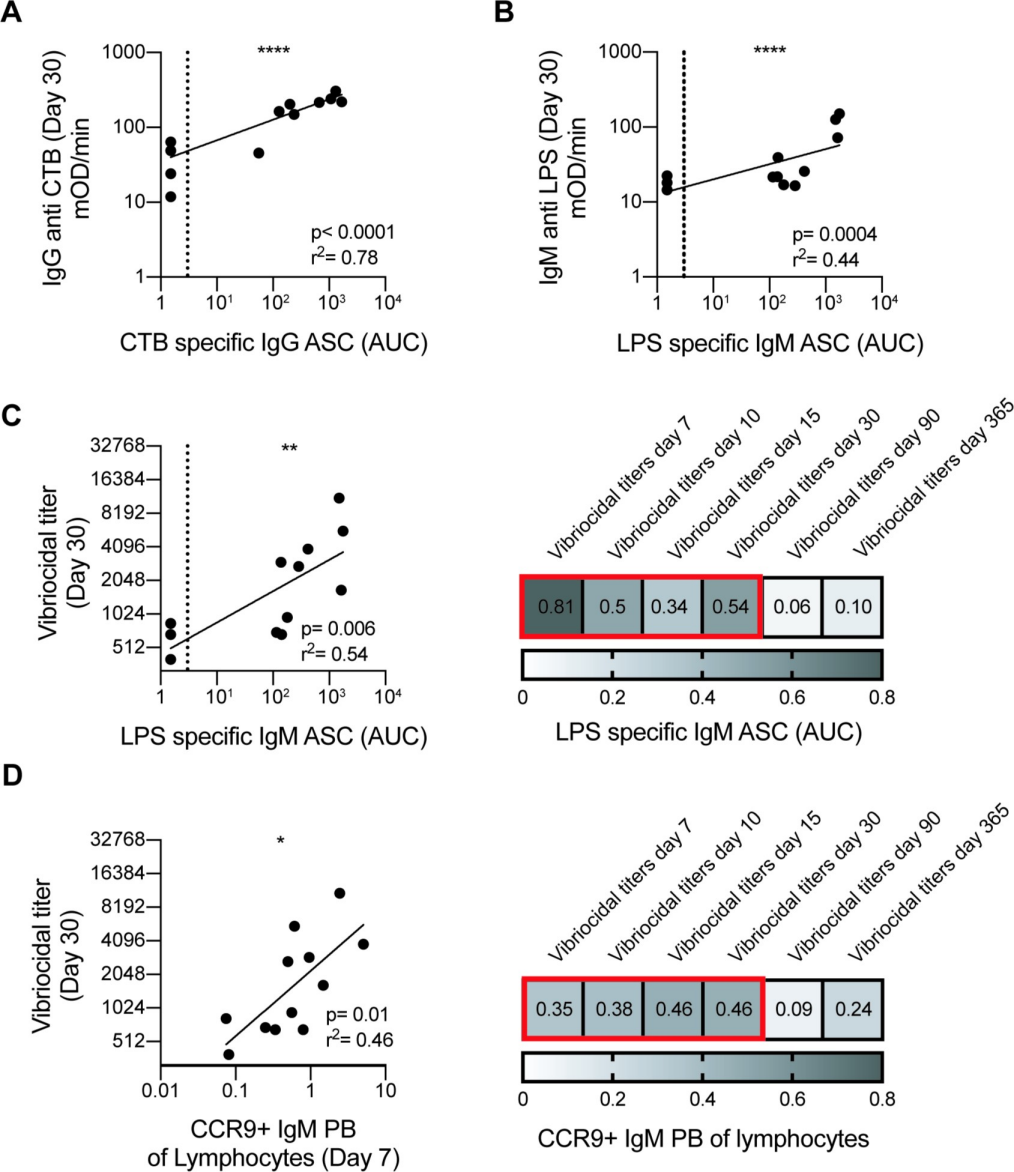
Correlations between early and late humoral responses. Linear regression analysis between (A) cholera toxin B subunit (CTB) specific IgG ASC and IgG CTB plasma titers at day 30 post vaccination. (B) Linear regression analysis between lipopolysaccharide (LPS) specific IgM ASC and IgM LPS plasma titers on day 30 post vaccination. (C) Representative (left panel) and summary (right panel) of linear regression analysis between LPS specific IgM ASC and vibriocidal plasma titers on day 30. Number in box is the r^2^ value and red outline indicates p<0.05. (D) Representative (left panel) and summary (right panel) linear regression analysis between CCR9+ IgM plasmablast of total lymphocytes and vibriocidal plasma titers. Number in box is the r^2^ value and red outline indicates p<0.05. ASC response for (C) and (D) is depicted as area under the curve (AUC) covering days 0, 7, 10, 15, and 30. Correlation analysis performed by log transformation of plasma titers and AUC followed by linear regression analysis. Dotted line represents the limit of detection for ELISPOT assay.

The main factor which drives humoral immunity to *V*. *cholerae* in humans has not yet been determined. Although it is not absolute, serum vibriocidal activity strongly correlates with protection [14]. Furthermore, little is known about the dynamics and durability of immune responses to a live attenuated cholera vaccine in an immunologically naïve setting, and how early responses may predict the outcome of immune parameters long term. As such, we attempted to correlate different early humoral responses measured at day 7 with the day 30, 90 and 365 vibriocidal titers to assess which parameters were predicative of persistent vibriocidal responses and thus potential long-term immunity. The antigen specific plasmablast responses at day 7 were predictive of the serological responses up to day 30 (**Fig 4A and 4B**), similar to what we have previously shown for influenza specific responses [38]. Of the early immune responses we examined, the percentage of IgM CCR9 expressing plasmablasts on day 7 as well as the amount of LPS specific IgM ASC showed the best correlation with early vibriocidal activity. Both the CCR9+ IgM plasmablast (r^2^ = 0.46 P = 0.05) and the LPS specific IgM ASC on day 7 (r^2^ = 0.54 P = 0.006) responses strongly predicted the vibriocidal activity at day 30 post vaccination **(Fig 4C and 4D)**. However, these correlations were no longer significant at the later day 90 and day 365 time points. In addition to these measures, we assessed additional parameters including but not limited to LPS specific and CTB specific serum titers, CTB specific IgM, IgG, and IgA ASC, MBC, total plasmablast magnitude, and agglutination. None of the aforementioned responses significantly predicted the long-term vibriocidal activity at day 90 or 365.

## Discussion

A thorough examination of the humoral immune response to *V*. *cholerae* infection is crucial to better understand protection against cholera. The live attenuated vaccine strain used in this study, Vaxchora or CVD-103 HgR, has been primarily used to protect individuals traveling to cholera endemic countries [9,24,26,39]. Thus many previous studies have primarily focused on clinical outcomes such as immunogenicity [29,40], safety [28], and protective efficacy [41]. In contrast, the current study focused on a detailed examination of the early immune response and how these early events correlate with long term-immunity. In addition, the study aimed to define the durability of immunity induced by this vaccine. With an obligate mucosal pathogen, homing to the intestinal mucosa is essential, and thus we also characterized the vaccine induced expression of the gut homing marker CCR9. Finally, as most cholera vaccine studies have been performed in highly endemic areas, much less is known about the primary immune responses against live bacteria. Taken together, this comprehensive analysis of the primary immune response after exposure to live bacteria in terms of the induction and durability of immunity after vaccination provides important insights into the induction of immunity against *V*. *cholerae* and should be valuable for ongoing and future vaccine development efforts.

Vibriocidal titers are a critical immune correlate of protection for cholera. Household contact studies in cholera endemic areas have reported that elevated vibriocidal titers inversely correlate with the incidence of infection [4,27,42]. Additionally, human challenge studies illustrate a positive correlation between vibriocidal titers and protection against severe disease [41]. The development of the peripheral vibriocidal titers reported in the present study are consistent with historical data of the humoral response to *V*. *cholerae* exposure. Studies of *V*. *cholerae* infection [43], and vaccination with CVD-103 HgR [44] or whole cell killed cholera vaccines [45] have all described the rapid increase and early peak of vibriocidal titers around 10 days post exposure with vibriocidal GMT ranging from of 4096–8192, 4314–4546, and around day 10 post exposure. Similar to those results, all participants in our study seroconverted by day 10 post vaccination. Given previously published findings in human challenge models [27], the magnitude of seroconversion observed in this study indicates that all participants would likely be protected against *V*. *cholerae* challenge for at least three months post vaccination. The vibriocidal titers declined from their peak at day 10 until they stabilized around 90 days post vaccination. Given the duration of these elevated titers, it is likely that many of our subjects would have some level of protection against challenge at later timepoints. Infection with *V*. *cholerae* is known to generate robust, durable immunity. When infected individuals were rechallenged 3 years following exposure, none of the subjects developed diarrhea, and only 50% had a detectable increase in vibriocidal titers [46]. It is possible that the response to the live attenuated vaccinate follows a similar trend. A study which examined the serological responses of subjects vaccinated by CVD-103 HgR when challenged 6 months post exposure showed that none of the subjects developed diarrhea, and approximately 50% - 73% of the participants had an increase in vibriocidal titers following challenge [41]. The vibriocidal titers measured prior to challenge ranged from GMT: 202 ~ 276, which is similar to what we observed in our cohort at one year post vaccination (GMT: 200) [41]. Given the similarity in titers, it is possible that our cohort would also remain protected against challenge at least up to 6 months post vaccination. Additionally, we observed that in our cohort the vibriocidal titers were stable up to one year post vaccination and the estimated half-life indicates that these titers would be able to persist at least up to two years post vaccination. A recent study supports this fact, as they were able to measure vibriocidal titers (GMT: 133) at 2 years post vaccination in a cohort of adolescents who had received the live attenuated vaccine [47]. While we are unable to predict the protective efficacy of the vaccine at these later timepoints, the persistence of these titers makes may indicate that these subjects would have some measure of immunity to exposure of wild type *V*. *cholerae* at these late timepoints.

Vibriocidal titers measure the ability of antibodies to bind to and induce complement mediated lysis of the bacteria. In the case of *V*. *cholerae*, this effect is primarily thought to be driven by LPS specific antibodies [21,48]. In concordance with prior infection [49] and CVD-103 HgR vaccine studies [28], we also saw a rapid increase in these titers following vaccination. In our vaccinees, the LPS specific serum titers and ASC responses primarily consisted of IgM and IgA antibodies. While LPS specific IgG antibodies have been detected in *V*. *cholerae* infected patients in endemic areas, the induction of these isotype switched antibodies appear to require either repeated exposure to the pathogen or a more severe infection to generate them [31,50,51]. Additionally, while IgG responses to LPS can occur their contribution to the overall vibriocidal activity, particularly in primary exposure, is uncertain. A recent study measured the contribution of IgM, and IgG, and IgA fractions to vibriocidal titers using serum collected from patients infected with *V*. *cholerae* [48]. Vibriocidal activity only significantly decreased when IgM was selectively depleted from the serum while no significant decrease in vibriocidal titers was observed when IgG or IgA was depleted. Those findings highlight the role that IgM antibodies have in driving peripheral vibriocidal titers. Likewise, the correlations performed in our study also show a strong link between vibriocidal titers and LPS specific IgM responses as measured by titers or early ASC. Therefore, due to the low amount of LPS specific IgG and the reduced ability of IgA antibodies to activate complement, the vibriocidal response in our cohort is likely predominantly mediated by LPS specific IgM antibodies.

In addition to LPS, a significant portion of the infection induced humoral antibody response targets the cholera toxin where it plays an important role in toxin neutralization [52–54]. Following immunization with the vaccine, between 50–80% of subjects will generate a fourfold or greater increase in anti-CTB titers following vaccination study [55,56]. Similar to those studies, we observed that our study cohort had seven members that had an increase in anti-CTB titers that was 4-fold or greater over day 0 titers at 30 days post vaccination. The total fold increase in anti-CTB IgG titers in our cohort was 4.7. The fold increase among the highest responders (4-fold or greater) was 6.8. This increase in magnitude is similar to what has previously reported by other groups following administration of the live attenuated vaccine, which ranges from a 4–16-fold increase in anti-CTB titers [57,58]. Additionally, the fold change in anti-CTB titers to the vaccine, particularly among the CTB responders, is similar to what has been observed following infection with wild type bacteria with an average increase between 4–12-fold [26,58,59]. In our study, most of the participants experienced a significant increase in antitoxin titers one-month post vaccination. However, in contrast to the LPS response, which was predominantly comprised of IgM and IgA antibodies, the cholera toxin response was largely derived from IgG and to a lesser extent IgA antibodies. As our cohort is primarily cholera naïve, it is surprising to see such a strong IgG response to the toxin antigen, given that isotype switched responses are generally associated with a secondary immune response. Cholera toxin is closely related to another diarrheal inducing agent, heat labile (LT) enterotoxin, which is produced by enterotoxigenic *E*. *coli (ETEC)*. LT, like cholera toxin, is an AB_5_ toxin, and binds to GM1 to enter target cells. The cholera toxin B subunit shares 83% homology with the B subunit of heat liable enterotoxin from ETEC [60,61]. Therefore, given the homology, it should be considered that the IgG class switched response observed in this study represents an anamnestic response driven by cells originally induced by prior exposure to LT. Indeed, a prior study from our lab has demonstrated that many of the CTB binding antibodies generated from infected patients are selective for LTB [21]. Finally, while *V*. *cholerae* infection is exceedingly rare within the United States, ETEC infections are far more common, both from cases of traveler’s diarrhea as well as domestic outbreaks. There were over 300 outbreaks of ETEC in the US alone between 2003–2012, with an estimated 35,000 cases annually, although this number is likely underreported [62,63].

When examining the antigen specific ASC responses, we observed that our cohort could be divided into three groups depending on their response to the cholera antigens. Those which had ASC responses either to LPS, CTB, or both antigens (**S5 Fig**). Of these three groups, participants which responded to both antigens tended to have the highest overall immune titers. The reason for this variation is unclear. In the case of the CTB response, individuals who had prior exposure to LTB may be able to generate higher responses to CTB compared to those who have not. When we examined this aspect in our cohort, we observed that subjects who had higher baseline titers to CTB on day 0 generally had a better response following vaccination (4-fold or greater increase in CTB IgG titers) to CTB compared to those that did not (**S6 Fig**). If the higher titers seen on day 0 are indicative to prior exposure to LTB, it could explain why these particular subjects generated a significant IgG response to CTB. Understanding the cause of these difference responses would be a key step to better understanding cholera immunity.

*V*. *cholerae* is a noninvasive pathogen and is thus not directly subjected to peripheral humoral responses. As the bacteria colonizes the small intestine it would primarily be subjected to antibodies that are secreted into the lumen. Therefore, examining the ASC which are responsible for producing the secreted antibodies is of particular interest. Plasmablasts are ASC that transiently circulate shortly after infection or vaccination [64]. As these cells represent an induced response, they are generally enriched for vaccine or pathogen specific ASCs. It has previously been demonstrated that plasmablasts are induced both by *V*. *cholerae* infection and vaccination [21,34]. Similar to those efforts, this study found that the plasmablast population peaked shortly after vaccination between days 7 and 10. In addition to the serological analysis, we found that many of the ASC in circulation were specific to the two major immunodominant antigens of cholera, LPS and cholera toxin. As *V*. *cholerae* is a mucosal pathogen, it is likely that a portion of the plasmablasts originate from mucosa associated lymphoid tissue (MALT) such as Peyer’s patches.

CCR9 is well described as a gut homing marker that promotes migration of cells to the small intestine by binding to CCL25 released from small intestinal epithelial cells [19,65]. Phenotypic characterization of the plasmablast population in our donors showed a significant increase in CCR9 expression following vaccination. Thus, it is likely that these ASC originated in the gut and have the potential to return there following systemic circulation. This increase in CCR9 expression was predominantly found on the IgM plasmablasts which were almost exclusively LPS specific by ELISPOT analysis. In addition to IgM, approximately 30% of the IgA plasmablasts also expressed CCR9. These two isotypes incorporate the J chain protein, which allows them to bind to the poly Ig receptor for transcytosis into the lumen by epithelial cells. Once in the lumen, the antibodies can mediate their effector functions through a variety of mechanisms.

In contrast to IgM, there was far less of an increase in CCR9 expression on the IgG secreting plasmablasts. As the IgG ASC response was almost exclusively CTB specific, it is possible that CTB does not result in the robust generation of CCR9 expressing IgG plasmablasts. There are a few possibilities that could account for this discrepancy in CCR9 expression such as the antigen, cytokines, and signals from other cells. Location of antigen uptake could also play a role given that CTB is secreted and could potentially end up in different anatomical sites that are more distal from the intestine as compared to the whole cell bacteria. This is particularly important given the role that location has in influencing the expression of homing receptors. For example, CCR9 expression on immune cells has been shown to be induced by the retinoic acid produced by the dendritic cells of gut associated lymphoid tissues (GALT) [66].Studies using mouse models have demonstrated that CCR9 expression on gut immune cells is heavily influenced by the draining lymph node the cell is activated in [67,68]. While the site of induction of immunity against cholera toxin in humans is not clear, a similar mechanism could explain the lack of CCR9+ IgG cells in our study.

The ability to predict the long-term effectiveness of cholera immunity using early makers of immunity is of particular interest. As vibriocidal activity is the main correlate of protection, we compared early makers of immunity such as plasma titers, ASC levels, and the magnitude and phenotype of responding plasmablasts to vibriocidal titers. Of the parameters examined, only the early LPS specific IgM titers, LPS specific IgM ASC, and CCR9 expressing IgM plasmablasts were all predictive of the with vibriocidal activity later in the immune response (day 30). However, the predictive value of these measure was no longer present systemically at day 90 or 365 post vaccination. Thus, the generation of long-lived antibody mediated immunity against cholera is regulated differently than the early responses, or the effector cells induced early in the response home to mucosal sites and are no longer detectable in circulation. Thus, it is conceivable that the early IgM ASC and CCR9+ plasmablast responses correlate better with long term immunity in the mucosa [65,69,70]. This is of particular interest given the high level of CCR9+ expression found on the IgM population, which was largely LPS specific. These issues are currently being investigated using small intestinal biopsies but are beyond the scope of the current study.

Our observations on the lack of correlation between the early plasmablast responses and the peripheral titers at one year post vaccination raises the question of what is responsible for driving the long-lived peripheral responses we detected in our study. Long-term protection immunity to *V*. *cholerae* is driven by LPS and CT specific antibodies. These long term antibody responses are thought to be maintained by long lived mucosal plasma cells [20], memory B cells [35], or a combination of the two. The CCR9+ plasmablasts seen in our cohort indicate the potential for long term mucosal plasma cell responses. There is also the possibility that the titers seen at 1 year are generated by bone marrow derived plasma cells. Future studies will direct efforts to understand the contribution of bone marrow and mucosal plasma cell compartments to peripheral titers. The degree to which peripheral immunity reflects mucosal immunity is the subject of many ongoing studies in the field of immunology. Understanding the link between the two locations will be critical for a better understanding of cholera immunity and guide ongoing vaccine development efforts.

The current study illustrates the value of the live attenuated *V*. *cholerae* vaccine as a model system to understand immunity against this pathogen in a setting of a primary exposure. Longer term studies using this system that incorporate repeated vaccination or challenge with virulent *V*. *cholerae* will provide the opportunity to better assess markers that are predictive of long-term immunity and identify the immunological mechanisms that result in the generation and maintenance of long-term protection. To address these questions, we are currently examining how the systemic response studied here correlates with mucosal responses measured in small intestinal biopsies. These efforts, in conjunction with comparative studies to recall responses in cholera endemic areas will provide additional insight into immunity against this pathogen. In conclusion, this study highlights key factors which may play an important role in the generation of immunity, such as the correlation between CCR9 and vibriocidal activity. Mediators of durable protective immunity may be identified through an iterative process of comparing immune response in both naïve and immune patients, which can lead to a better understanding of the mucosal immune response to both this disease and other mucosal infections as well as empower ongoing vaccine development strategies.

## Supporting information

S1 Fig Antibodies from vaccinated donors are able to bind to the cholera holotoxin. Summary ELISPOT analysis of CTB and CTH specific IgM, IgG, and IgA antibody secreting cells measured on day 7 post vaccination for each of the 12 participants. Dotted line indicates limit of detection of the ELISPOT assay.
(TIF)

S2 Fig Kinetic analysis of antigen specific ASC following vaccination. Summary ELISPOT analysis of lipopolysaccharide (LPS) and cholera toxin B subunit (CTB) specific IgM, IgG, and IgA antibody secreting cells following vaccination on day 0, 7, 10, 15 and 30 post vaccination from each of the 12 subjects. Dotted line indicates limit of detection of the ELISPOT assay.
(TIF)

S3 Fig ASC responses are cross reactive with LPS from *V. cholerae* serotype Ogawa. Summary ELISPOT analysis of Inaba and Ogawa LPS specific IgM, IgG, and IgA antibody secreting cells following vaccination from each of the 12 subjects on day 7 post vaccination. Dashed line indicates limit of detection of the ELISPOT assay.
(TIF)

S4 Fig Memory B cell responses are modestly elevated post vaccination. (A) Representative ELISPOT and summary analysis of (B) LPS and (C) CTB specific IgG and IgA memory B cells on days 0, 30, and 90 post vacation for each of the 12 participants. Dashed line indicates limit of detection for the memory B cell assay.
(TIF)

S5 Fig Antigen specific donor responses. All 12 subjects are divided into LPS only, CTB only, and LPS/CTB double responders based on antibody secreting cell responses to the immunodominant cholera antigens LPS and CTB. (A) Day 7 ASC responses from each subject to LPS and CTB as measured by ELISPOT. (B) Percentage of plasmablasts which were CCR9+ as measured by flow cytometry on 7 days post vaccination for each of the study participants. (C) Vibriocidal titers as measured on day 0, 10, and 90 for each participant. (D) Agglutination titer measured on day 0, 10, and 90 for each participant. (E) IgM anti LPS titers measured on day 0, 10, and 90 for each subject. (F) IgG anti CTB titers measured on day 0, 10, and 90 for each subject.
(TIF)

S6 Fig High baseline CTB titers correlate with greater response to CTB. (A) Anti IgG CTB titers as measured by ELISA on day 0 and 30 post vaccination for each subject. (B) Linear regression analysis of IgG anti CTB titers as measured by ELISA on day 0 (x axis) and day 30 (y axis) post vaccination. Significance values are indicated by asterisks (P < 0.05 (*); P < 0.005 (**); P < 0.0005 (***); P <0.0001 (****)).
(TIF)

## References

[1] Ali M, Nelson AR, Lopez AL, Sack DA. Updated Global Burden of Cholera in Endemic Countries. Plos Neglect Trop D. 2015;9(6). ARTN e0003832; doi: 10.1371/journal.pntd.0003832 WOS:000357398100034; PubMed Central PMCID: PMC4455997. 26043000 PMC4455997

[2] Qadri F, Islam T, Clemens JD. Cholera in Yemen—An Old Foe Rearing Its Ugly Head. N Engl J Med. 2017. Epub 2017/11/02. doi: 10.1056/NEJMp1712099. 29091747

[3] Santé WHOOmdl. Weekly Epidemiological Record. Weekly Epidemiological Record = Relevé épidémiologique hebdomadaire. 2020; 95 (37)(37): 441–8. Epub 11 September 2020.

[4] Mosley WH, Ahmad S, Benenson AS, Ahmed A. The relationship of vibriocidal antibody titre to susceptibility to cholera in family contacts of cholera patients. Bull World Health Organ. 1968;38(5):777–85. ; PubMed Central PMCID: PMC2554681. 5303331 PMC2554681

[5] Mosley WH, McCormack WM, Ahmed A, Chowdhury AK, Barui RK. Report of the 1966–67 cholera vaccine field trial in rural East Pakistan. 2. Results of the serological surveys in the study population—the relationship of case rate to antibody titre and an estimate of the inapparent infection rate with Vibrio cholerae. Bull World Health Organ. 1969;40(2):187–97. ; PubMed Central PMCID: PMC2554605. 5306539 PMC2554605

[6] Glass RI, Svennerholm AM, Khan MR, Huda S, Huq MI, Holmgren J. Seroepidemiological Studies of El-Tor Cholera in Bangladesh—Association of Serum Antibody-Levels with Protection. Journal of Infectious Diseases. 1985;151(2):236–42. WOS:A1985AAS1200006. doi: 10.1093/infdis/151.2.236 3968450

[7] Kabir S. Critical analysis of compositions and protective efficacies of oral killed cholera vaccines. Clin Vaccine Immunol. 2014;21(9):1195–205. Epub e. doi: 10.1128/CVI.00378-14 ; PubMed Central PMCID: PMC4178583. 25056361 PMC4178583

[8] Wang Z, Lazinski DW, Camilli A. Immunity Provided by an Outer Membrane Vesicle Cholera Vaccine Is Due to O-Antigen-Specific Antibodies Inhibiting Bacterial Motility. Infect Immun. 2017;85(1). doi: 10.1128/IAI.00626-16; PubMed Central PMCID: PMC5203661. 27795359 PMC5203661

[9] Levine MM, Chen WH, Kaper JB, Lock M, Danzig L, Gurwith M. PaxVax CVD 103-HgR single-dose live oral cholera vaccine. Expert Rev Vaccines. 2017;16(3):197–213. doi: 10.1080/14760584.2017.1291348 . 28165831

[10] Teoh SL, Kotirum S, Hutubessy R, Chaiyakunapruk N. Global Economic Evaluation of Oral Cholera Vaccine: A Systematic Review. Hum Vaccin Immunother. 2017:0. Epub 2017/11/04. doi: 10.1080/21645515.2017.1392422. 29099647 PMC5806687

[11] Clemens JD, Nair GB, Ahmed T, Qadri F, Holmgren J. Cholera. Lancet. 2017;390(10101):1539–49. Epub 2017/03/18. doi: 10.1016/S0140-6736(17)30559-7 . 28302312

[12] Lippi D, Gotuzzo E, Caini S. Cholera. Microbiol Spectr. 2016;4(4). Epub 2016/10/12. doi: 10.1128/microbiolspec.PoH-0012-2015. 27726771

[13] Benenson AS, Saad A, Mosley WH. Serological studies in cholera. 2. The vibriocidal antibody response of cholera patients determined by a microtechnique. Bull World Health Organ. 1968;38(2):277–85. ; PubMed Central PMCID: PMC2554323. 5302303 PMC2554323

[14] Levine MM, Nalin DR, Craig JP, Hoover D, Bergquist EJ, Waterman D, et al. Immunity of cholera in man: relative role of antibacterial versus antitoxic immunity. Trans R Soc Trop Med Hyg. 1979;73(1):3–9. doi: 10.1016/0035-9203(79)90119-6 . 442179

[15] Saha D, LaRocque RC, Khan AI, Harris JB, Begum YA, Akramuzzaman SM, et al. Incomplete correlation of serum vibriocidal antibody titer with protection from Vibrio cholerae infection in urban Bangladesh. J Infect Dis. 2004;189(12):2318–22. doi: 10.1086/421275 . 15181581

[16] Sina C, Kemper C, Derer S. The intestinal complement system in inflammatory bowel disease: Shaping intestinal barrier function. Semin Immunol. 2018;37:66–73. Epub 2018/03/01. doi: 10.1016/j.smim.2018.02.008 . 29486961

[17] Kopp ZA, Jain U, Van Limbergen J, Stadnyk AW. Do antimicrobial peptides and complement collaborate in the intestinal mucosa? Front Immunol. 2015;6:17. doi: 10.3389/fimmu.2015.00017; PubMed Central PMCID: PMC4311685. 25688244 PMC4311685

[18] Jain U, Otley AR, Van Limbergen J, Stadnyk AW. The complement system in inflammatory bowel disease. Inflamm Bowel Dis. 2014;20(9):1628–37. Epub 2014/05/17. doi: 10.1097/MIB.0000000000000056 . 24831561

[19] Spencer J, Sollid LM. The human intestinal B-cell response. Mucosal Immunol. 2016;9(5):1113–24. Epub 2016/07/28. doi: 10.1038/mi.2016.59 . 27461177

[20] Uddin T, Harris JB, Bhuiyan TR, Shirin T, Uddin MI, Khan AI, et al. Mucosal immunologic responses in cholera patients in Bangladesh. Clin Vaccine Immunol. 2011;18(3):506–12. doi: 10.1128/CVI.00481-10 ; PubMed Central PMCID: PMC3067383. 21248157 PMC3067383

[21] Kauffman RC, Bhuiyan TR, Nakajima R, Mayo-Smith LM, Rashu R, Hoq MR, et al. Single-Cell Analysis of the Plasmablast Response to Vibrio cholerae Demonstrates Expansion of Cross-Reactive Memory B Cells. MBio. 2016;7(6). doi: 10.1128/mBio.02021-16; PubMed Central PMCID: PMC5181778. 27999163 PMC5181778

[22] Priyamvada L, Cho A, Onlamoon N, Zheng NY, Huang M, Kovalenkov Y, et al. B Cell Responses during Secondary Dengue Virus Infection Are Dominated by Highly Cross-Reactive, Memory-Derived Plasmablasts. J Virol. 2016;90(12):5574–85. doi: 10.1128/JVI.03203-15 ; PubMed Central PMCID: PMC4886779. 27030262 PMC4886779

[23] Priyamvada L, Quicke KM, Hudson WH, Onlamoon N, Sewatanon J, Edupuganti S, et al. Human antibody responses after dengue virus infection are highly cross-reactive to Zika virus. Proc Natl Acad Sci U S A. 2016;113(28):7852–7. doi: 10.1073/pnas.1607931113 ; PubMed Central PMCID: PMC4948328. 27354515 PMC4948328

[24] Herzog C. Successful comeback of the single-dose live oral cholera vaccine CVD 103-HgR. Travel Med Infect Dis. 2016;14(4):373–7. doi: 10.1016/j.tmaid.2016.07.003 . 27425792

[25] Kaper JB, Lockman H, Baldini MM, Levine MM. A Recombinant Live Oral Cholera Vaccine. Bio/Technology. 1984;2(4):345–9. doi: 10.1038/nbt0484-345

[26] Levine MM, Kaper JB, Herrington D, Ketley J, Losonsky G, Tacket CO, et al. Safety, immunogenicity, and efficacy of recombinant live oral cholera vaccines, CVD 103 and CVD 103-HgR. Lancet. 1988;2(8609):467–70. doi: 10.1016/s0140-6736(88)90120-1 . 2900401

[27] Chen WH, Cohen MB, Kirkpatrick BD, Brady RC, Galloway D, Gurwith M, et al. Single-dose Live Oral Cholera Vaccine CVD 103-HgR Protects Against Human Experimental Infection With Vibrio cholerae O1 El Tor. Clin Infect Dis. 2016;62(11):1329–35. doi: 10.1093/cid/ciw145 ; PubMed Central PMCID: PMC4872293. 27001804 PMC4872293

[28] Chen WH, Greenberg RN, Pasetti MF, Livio S, Lock M, Gurwith M, et al. Safety and immunogenicity of single-dose live oral cholera vaccine strain CVD 103-HgR, prepared from new master and working cell banks. Clin Vaccine Immunol. 2014;21(1):66–73. doi: 10.1128/CVI.00601-13 ; PubMed Central PMCID: PMC3910924. 24173028 PMC3910924

[29] Su-Arehawaratana P, Singharaj P, Taylor DN, Hoge C, Trofa A, Kuvanont K, et al. Safety and immunogenicity of different immunization regimens of CVD 103-HgR live oral cholera vaccine in soldiers and civilians in Thailand. J Infect Dis. 1992;165(6):1042–8. doi: 10.1093/infdis/165.6.1042 . 1583321

[30] Crotty S, Aubert RD, Glidewell J, Ahmed R. Tracking human antigen-specific memory B cells: a sensitive and generalized ELISPOT system. J Immunol Methods. 2004;286(1–2):111–22. Epub 2004/04/17. doi: 10.1016/j.jim.2003.12.015 . 15087226

[31] Aktar A, Rahman MA, Afrin S, Faruk MO, Uddin T, Akter A, et al. O-Specific Polysaccharide-Specific Memory B Cell Responses in Young Children, Older Children, and Adults Infected with Vibrio cholerae O1 Ogawa in Bangladesh. Clin Vaccine Immunol. 2016;23(5):427–35. doi: 10.1128/CVI.00647-15 ; PubMed Central PMCID: PMC4860469. 27009211 PMC4860469

[32] Champsaur H, Fattal-German M, Arranhado R. Sensitivity and specificity of viral immunoglobulin M determination by indirect enzyme-linked immunosorbent assay. J Clin Microbiol. 1988;26(2):328–32. Epub 1988/02/01. doi: 10.1128/jcm.26.2.328-332.1988 ; PubMed Central PMCID: PMC266277. 3125220 PMC266277

[33] Pyndiah N, Krech U, Price P, Wilhelm J. Simplified chromatographic separation of immunoglobulin M from G and its application to toxoplasma indirect immunofluorescence. J Clin Microbiol. 1979;9(2):170–4. Epub 1979/02/01. doi: 10.1128/jcm.9.2.170-174.1979 ; PubMed Central PMCID: PMC272984. 372221 PMC272984

[34] Rahman A, Rashu R, Bhuiyan TR, Chowdhury F, Khan AI, Islam K, et al. Antibody-secreting cell responses after Vibrio cholerae O1 infection and oral cholera vaccination in adults in Bangladesh. Clin Vaccine Immunol. 2013;20(10):1592–8. doi: 10.1128/CVI.00347-13 ; PubMed Central PMCID: PMC3807192. 23945156 PMC3807192

[35] Patel SM, Rahman MA, Mohasin M, Riyadh MA, Leung DT, Alam MM, et al. Memory B cell responses to Vibrio cholerae O1 lipopolysaccharide are associated with protection against infection from household contacts of patients with cholera in Bangladesh. Clin Vaccine Immunol. 2012;19(6):842–8. doi: 10.1128/CVI.00037-12 ; PubMed Central PMCID: PMC3370438. 22518009 PMC3370438

[36] Leung DT, Rahman MA, Mohasin M, Patel SM, Aktar A, Khanam F, et al. Memory B cell and other immune responses in children receiving two doses of an oral killed cholera vaccine compared to responses following natural cholera infection in Bangladesh. Clin Vaccine Immunol. 2012;19(5):690–8. Epub 2012/03/24. doi: 10.1128/CVI.05615-11 ; PubMed Central PMCID: PMC3346319. 22441386 PMC3346319

[37] Weisel NM, Weisel FJ, Farber DL, Borghesi LA, Shen Y, Ma W, et al. Comprehensive analyses of B-cell compartments across the human body reveal novel subsets and a gut-resident memory phenotype. Blood. 2020;136(24):2774–85. Epub 2020/08/05. doi: 10.1182/blood.2019002782 ; PubMed Central PMCID: PMC7731793. 32750113 PMC7731793

[38] Cho A, Bradley B, Kauffman R, Priyamvada L, Kovalenkov Y, Feldman R, et al. Robust memory responses against influenza vaccination in pemphigus patients previously treated with rituximab. JCI Insight. 2017;2(12). Epub 2017/06/15. doi: 10.1172/jci.insight.93222; PubMed Central PMCID: PMC5470882. 28614800 PMC5470882

[39] Cabrera A, Lepage JE, Sullivan KM, Seed SM. Vaxchora: A Single-Dose Oral Cholera Vaccine. Ann Pharmacother. 2017;51(7):584–9. Epub 2017/06/18. doi: 10.1177/1060028017698162 . 28622736

[40] Suharyono, Simanjuntak C, Totosudirjo H, Witham N, Punjabi N, Burr D, et al. Safety and immunogenicity of single-dose live oral cholera vaccine CVD 103-HgR in 5-9-year-old Indonesian children. The Lancet. 1992;340(8821):689–94. doi: 10.1016/0140-6736(92)92231-4 1355798

[41] Tacket CO, Losonsky G, Nataro JP, Cryz SJ, Edelman R, Kaper JB, et al. Onset and duration of protective immunity in challenged volunteers after vaccination with live oral cholera vaccine CVD 103-HgR. J Infect Dis. 1992;166(4):837–41. doi: 10.1093/infdis/166.4.837 . 1527420

[42] Clemens JD, van Loon F, Sack DA, Chakraborty J, Rao MR, Ahmed F, et al. Field trial of oral cholera vaccines in Bangladesh: serum vibriocidal and antitoxic antibodies as markers of the risk of cholera. J Infect Dis. 1991;163(6):1235–42. doi: 10.1093/infdis/163.6.1235 . 2037789

[43] Harris AM, Bhuiyan MS, Chowdhury F, Khan AI, Hossain A, Kendall EA, et al. Antigen-specific memory B-cell responses to Vibrio cholerae O1 infection in Bangladesh. Infect Immun. 2009;77(9):3850–6. doi: 10.1128/IAI.00369-09 ; PubMed Central PMCID: PMC2738048. 19528207 PMC2738048

[44] Wasserman SS, Losonsky GA, Noriega F, Tacket CO, Castaneda E, Levine MM. Kinetics of the vibriocidal antibody response to live oral cholera vaccines. Vaccine. 1994;12(11):1000–3. doi: 10.1016/0264-410x(94)90335-2 . 7975839

[45] Alam MM, Riyadh MA, Fatema K, Rahman MA, Akhtar N, Ahmed T, et al. Antigen-specific memory B-cell responses in Bangladeshi adults after one- or two-dose oral killed cholera vaccination and comparison with responses in patients with naturally acquired cholera. Clin Vaccine Immunol. 2011;18(5):844–50. doi: 10.1128/CVI.00562-10 ; PubMed Central PMCID: PMC3122537. 21346055 PMC3122537

[46] Levine MM, Black RE, Clements ML, Cisneros L, Nalin DR, Young CR. Duration of infection-derived immunity to cholera. J Infect Dis. 1981;143(6):818–20. doi: 10.1093/infdis/143.6.818 . 7252264

[47] McCarty JM, Cassie D, Bedell L, Lock MD, Bennett S. Long-Term Immunogenicity of Live Oral Cholera Vaccine CVD 103-HgR in Adolescents Aged 12–17 Years in the United States. Am J Trop Med Hyg. 2021. Epub 2021/04/06. doi: 10.4269/ajtmh.20-1576; PubMed Central PMCID: PMC8103473. 33819178 PMC8103473

[48] Yang JS, An SJ, Jang MS, Song M, Han SH. IgM specific to lipopolysaccharide of Vibrio cholerae is a surrogate antibody isotype responsible for serum vibriocidal activity. PLoS One. 2019;14(3):e0213507. Epub 2019/03/08. doi: 10.1371/journal.pone.0213507; PubMed Central PMCID: PMC6405115. 30845262 PMC6405115

[49] Sack RB, Barua D, Saxena R, Carpenter CC. Vibriocidal and agglutinating antibody patterns in cholera patients. J Infect Dis. 1966;116(5):630–40. Epub 1966/12/01. doi: 10.1093/infdis/116.5.630 . 5957269

[50] Johnson RA, Uddin T, Aktar A, Mohasin M, Alam MM, Chowdhury F, et al. Comparison of immune responses to the O-specific polysaccharide and lipopolysaccharide of Vibrio cholerae O1 in Bangladeshi adult patients with cholera. Clin Vaccine Immunol. 2012;19(11):1712–21. doi: 10.1128/CVI.00321-12 ; PubMed Central PMCID: PMC3491541. 22993410 PMC3491541

[51] Uddin T, Aktar A, Xu P, Johnson RA, Rahman MA, Leung DT, et al. Immune responses to O-specific polysaccharide and lipopolysaccharide of Vibrio cholerae O1 Ogawa in adult Bangladeshi recipients of an oral killed cholera vaccine and comparison to responses in patients with cholera. Am J Trop Med Hyg. 2014;90(5):873–81. doi: 10.4269/ajtmh.13-0498 ; PubMed Central PMCID: PMC4015581. 24686738 PMC4015581

[52] Herrington DA, Hall RH, Losonsky G, Mekalanos JJ, Taylor RK, Levine MM. Toxin, toxin-coregulated pili, and the toxR regulon are essential for Vibrio cholerae pathogenesis in humans. J Exp Med. 1988;168(4):1487–92. Epub 1988/10/01. doi: 10.1084/jem.168.4.1487 ; PubMed Central PMCID: PMC2189073. 2902187 PMC2189073

[53] Elson CO, Ealding W. Generalized systemic and mucosal immunity in mice after mucosal stimulation with cholera toxin. J Immunol. 1984;132(6):2736–41. Epub 1984/06/01. . 6233359

[54] Cash RA, Music SI, Libonati JP, Snyder MJ, Wenzel RP, Hornick RB. Response of man to infection with Vibrio cholerae. I. Clinical, serologic, and bacteriologic responses to a known inoculum. J Infect Dis. 1974;129(1):45–52. Epub 1974/01/01. doi: 10.1093/infdis/129.1.45 . 4809112

[55] Tacket CO, Cohen MB, Wasserman SS, Losonsky G, Livio S, Kotloff K, et al. Randomized, double-blind, placebo-controlled, multicentered trial of the efficacy of a single dose of live oral cholera vaccine CVD 103-HgR in preventing cholera following challenge with Vibrio cholerae O1 El tor inaba three months after vaccination. Infect Immun. 1999;67(12):6341–5. doi: 10.1128/IAI.67.12.6341-6345.1999 ; PubMed Central PMCID: PMC97039. 10569747 PMC97039

[56] Kotloff KL, Wasserman SS, O’Donnell S, Losonsky GA, Cryz SJ, Levine MM. Safety and immunogenicity in North Americans of a single dose of live oral cholera vaccine CVD 103-HgR: results of a randomized, placebo-controlled, double-blind crossover trial. Infect Immun. 1992;60(10):4430–2. Epub 1992/10/01. doi: 10.1128/iai.60.10.4430-4432.1992 ; PubMed Central PMCID: PMC257485. 1398956 PMC257485

[57] Haney DJ, Lock MD, Simon JK, Harris J, Gurwith M. Antibody-Based Correlates of Protection Against Cholera Analysis of a Challenge Study in a Cholera-Naive Population. Clin Vaccine Immunol. 2017. doi: 10.1128/CVI.00098-17; PubMed Central PMCID: PMC5583470. 28566334 PMC5583470

[58] Mayo-Smith LM, Simon JK, Chen WH, Haney D, Lock M, Lyon CE, et al. The Live Attenuated Cholera Vaccine CVD 103-HgR Primes Responses to the Toxin-Coregulated Pilus Antigen TcpA in Subjects Challenged with Wild-Type Vibrio cholerae. Clin Vaccine Immunol. 2017;24(1). Epub 2016/11/17. doi: 10.1128/CVI.00470-16; PubMed Central PMCID: PMC5216439. 27847368 PMC5216439

[59] Losonsky GA, Tacket CO, Wasserman SS, Kaper JB, Levine MM. Secondary Vibrio cholerae-specific cellular antibody responses following wild-type homologous challenge in people vaccinated with CVD 103-HgR live oral cholera vaccine: changes with time and lack of correlation with protection. Infect Immun. 1993;61(2):729–33. doi: 10.1128/iai.61.2.729-733.1993 ; PubMed Central PMCID: PMC302786. 8423098 PMC302786

[60] Dallas WS, Falkow S. Amino acid sequence homology between cholera toxin and Escherichia coli heat-labile toxin. Nature. 1980;288(5790):499–501. Epub 1980/12/04. doi: 10.1038/288499a0 . 7003397

[61] Domenighini M, Pizza M, Jobling MG, Holmes RK, Rappuoli R. Identification of errors among database sequence entries and comparison of correct amino acid sequences for the heat-labile enterotoxins of Escherichia coli and Vibrio cholerae. Mol Microbiol. 1995;15(6):1165–7. Epub 1995/03/01. doi: 10.1111/j.1365-2958.1995.tb02289.x . 7623669

[62] Heiman KE, Mody RK, Johnson SD, Griffin PM, Gould LH. Escherichia coli O157 Outbreaks in the United States, 2003–2012. Emerg Infect Dis. 2015;21(8):1293–301. Epub 2015/07/23. doi: 10.3201/eid2108.141364 ; PubMed Central PMCID: PMC4517704. 26197993 PMC4517704

[63] Scallan E, Hoekstra RM, Angulo FJ, Tauxe RV, Widdowson MA, Roy SL, et al. Foodborne illness acquired in the United States—major pathogens. Emerg Infect Dis. 2011;17(1):7–15. Epub 2011/01/05. doi: 10.3201/eid1701.p11101 ; PubMed Central PMCID: PMC3375761. 21192848 PMC3375761

[64] Jackson SM, Wilson PC, James JA, Capra JD. Human B cell subsets. Adv Immunol. 2008;98:151–224. Epub 2008/09/06. doi: 10.1016/S0065-2776(08)00405-7 . 18772006

[65] Habtezion A, Nguyen LP, Hadeiba H, Butcher EC. Leukocyte Trafficking to the Small Intestine and Colon. Gastroenterology. 2016;150(2):340–54. Epub 2015/11/10. doi: 10.1053/j.gastro.2015.10.046 ; PubMed Central PMCID: PMC4758453. 26551552 PMC4758453

[66] Mora JR, Iwata M, Eksteen B, Song SY, Junt T, Senman B, et al. Generation of gut-homing IgA-secreting B cells by intestinal dendritic cells. Science. 2006;314(5802):1157–60. Epub 2006/11/18. doi: 10.1126/science.1132742 . 17110582

[67] Hammerschmidt SI, Ahrendt M, Bode U, Wahl B, Kremmer E, Forster R, et al. Stromal mesenteric lymph node cells are essential for the generation of gut-homing T cells in vivo. J Exp Med. 2008;205(11):2483–90. Epub 2008/10/15. doi: 10.1084/jem.20080039 ; PubMed Central PMCID: PMC2571923. 18852290 PMC2571923

[68] Houston SA, Cerovic V, Thomson C, Brewer J, Mowat AM, Milling S. The lymph nodes draining the small intestine and colon are anatomically separate and immunologically distinct. Mucosal Immunol. 2016;9(2):468–78. Epub 2015/09/04. doi: 10.1038/mi.2015.77 . 26329428

[69] Berkowska MA, Schickel JN, Grosserichter-Wagener C, de Ridder D, Ng YS, van Dongen JJ, et al. Circulating Human CD27-IgA+ Memory B Cells Recognize Bacteria with Polyreactive Igs. J Immunol. 2015;195(4):1417–26. doi: 10.4049/jimmunol.1402708 ; PubMed Central PMCID: PMC4595932. 26150533 PMC4595932

[70] Kim L, Martinez CJ, Hodgson KA, Trager GR, Brandl JR, Sandefer EP, et al. Systemic and mucosal immune responses following oral adenoviral delivery of influenza vaccine to the human intestine by radio controlled capsule. Sci Rep. 2016;6:37295. doi: 10.1038/srep37295; PubMed Central PMCID: PMC5121599 Vaxart, the sponsor of the study. The authors EPS and WJD work for a contract research organization that ran the study for the sponsor. 27881837 PMC5121599

