# Supplementary figures and images for: Longitudinal analysis of human humoral responses after vaccination with a live attenuated V. cholerae vaccine
Source: PLoS Negl Trop Dis. 2021 Sep 3;15(9):e0009743. doi: 10.1371/journal.pntd.0009743 (PMC8445443; doi:10.1371/journal.pntd.0009743)

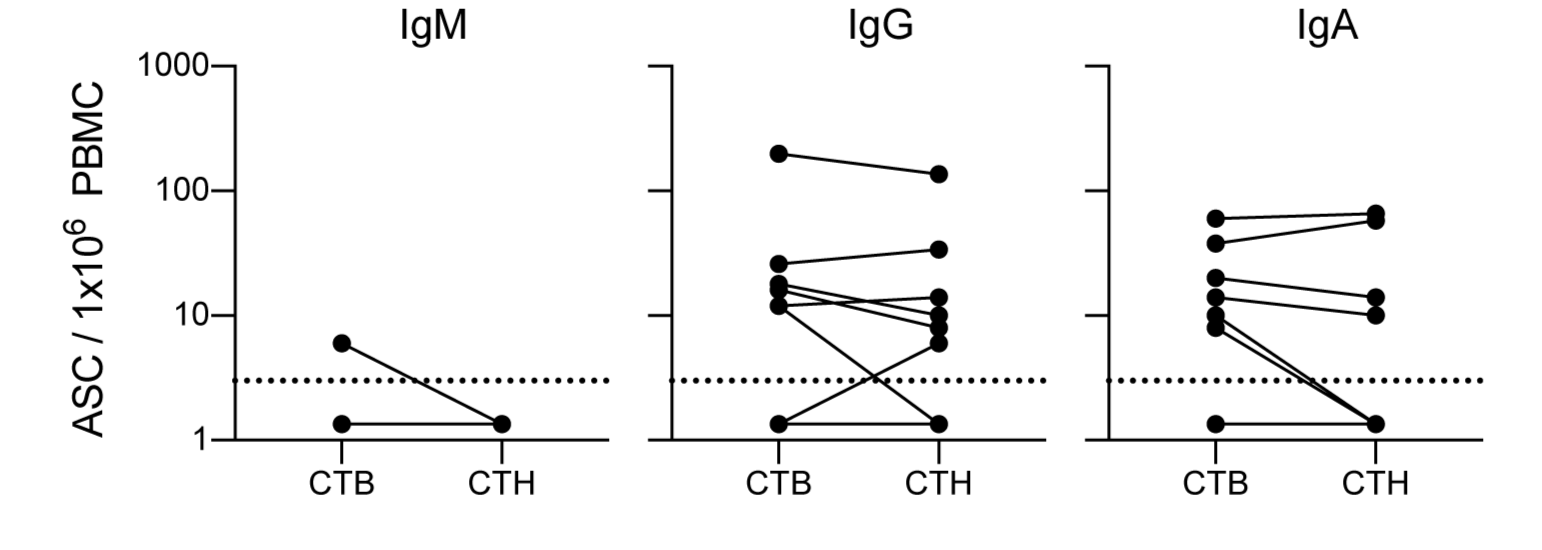

Supplement: S1 Fig — Summary ELISPOT analysis of CTB and CTH specific IgM, IgG, and IgA antibody secreting cells measured on day 7 post vaccination for each of the 12 participants. Dotted line indicates limit of detection of the ELISPOT assay. (TIF) [file pntd.0009743.s001.tif]

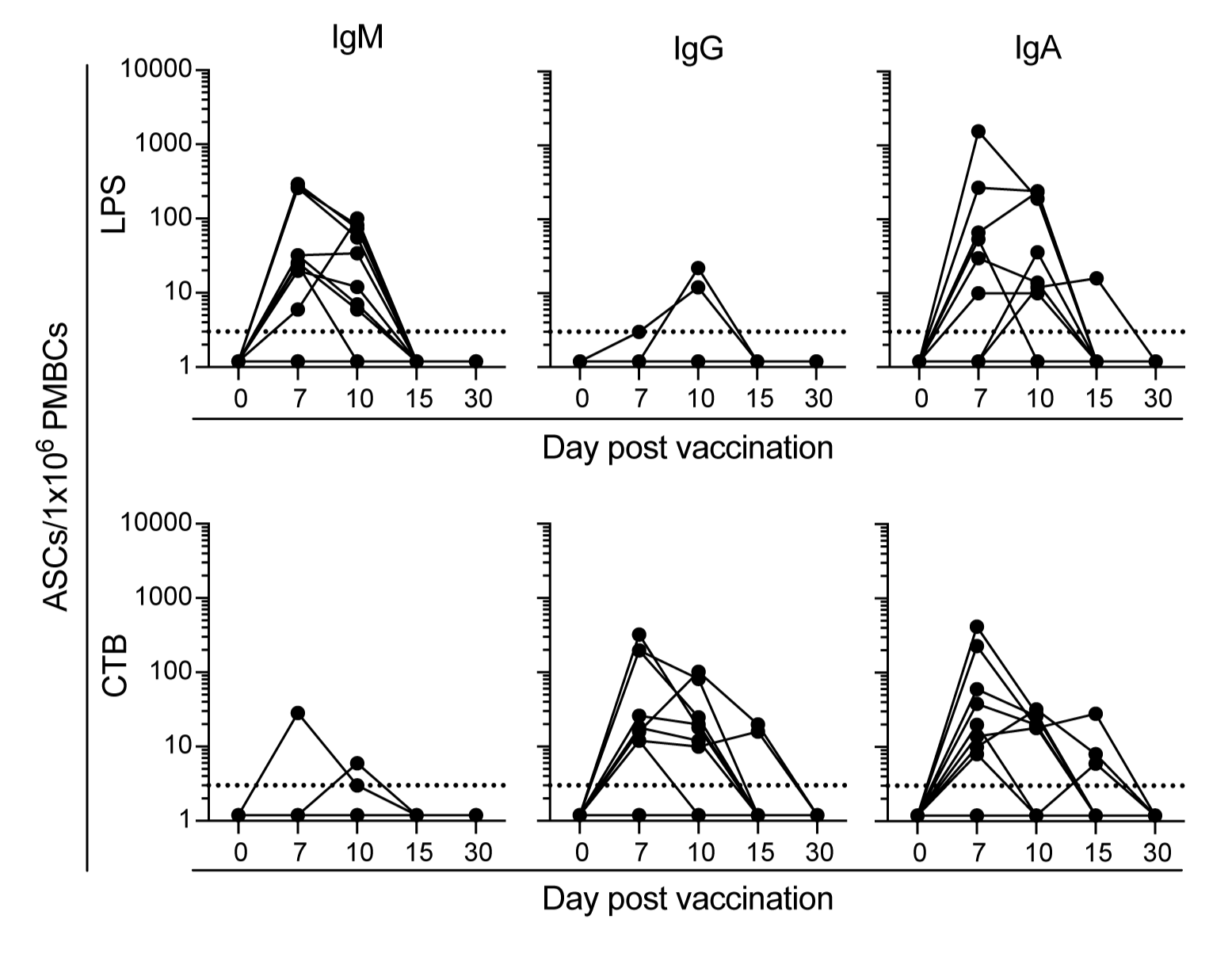

Supplement: S2 Fig — Summary ELISPOT analysis of lipopolysaccharide (LPS) and cholera toxin B subunit (CTB) specific IgM, IgG, and IgA antibody secreting cells following vaccination on day 0, 7, 10, 15 and 30 post vaccination from each of the 12 subjects. Dotted line indicates limit of detection of the ELISPOT assay. (TIF) [file pntd.0009743.s002.tif]

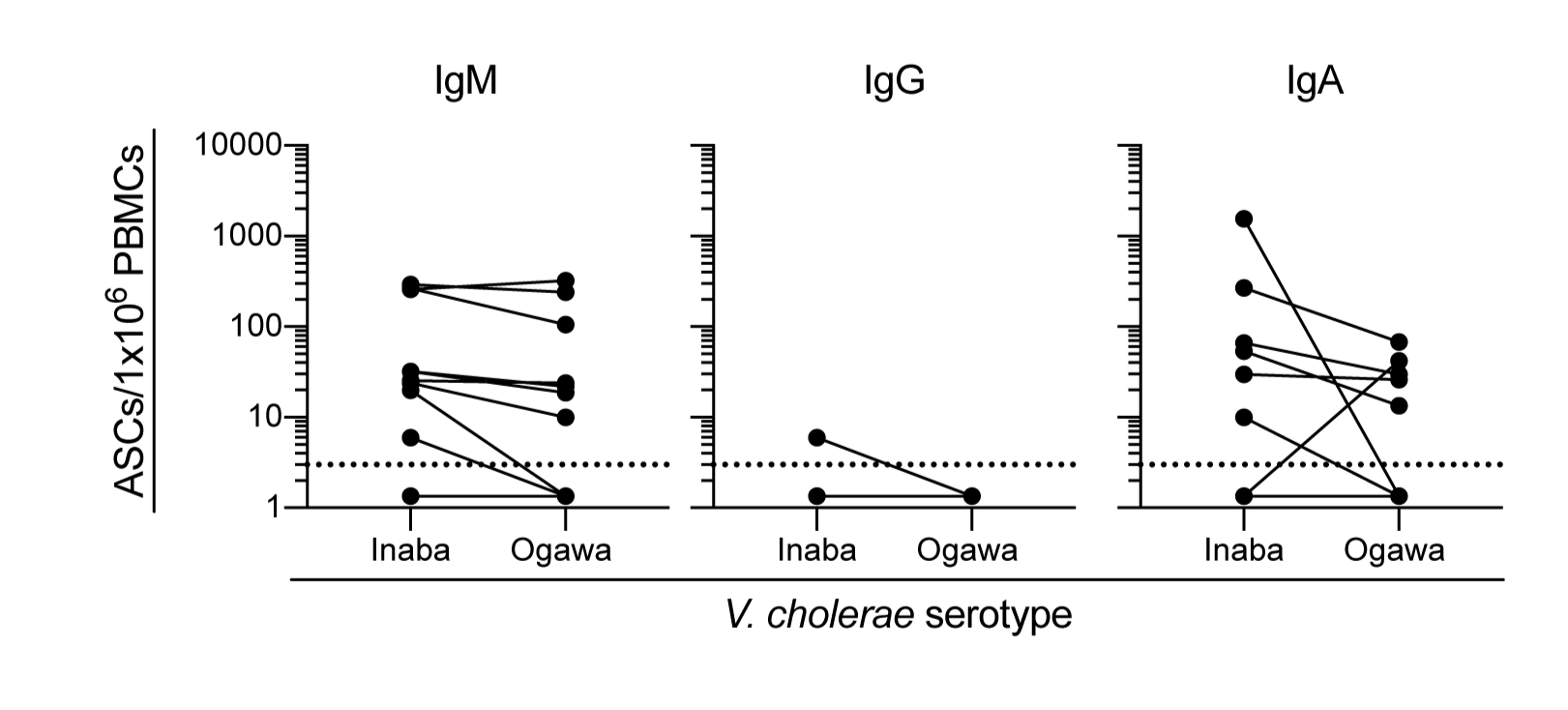

Supplement: S3 Fig — Summary ELISPOT analysis of Inaba and Ogawa LPS specific IgM, IgG, and IgA antibody secreting cells following vaccination from each of the 12 subjects on day 7 post vaccination. Dashed line indicates limit of detection of the ELISPOT assay. (TIF) [file pntd.0009743.s003.tif]

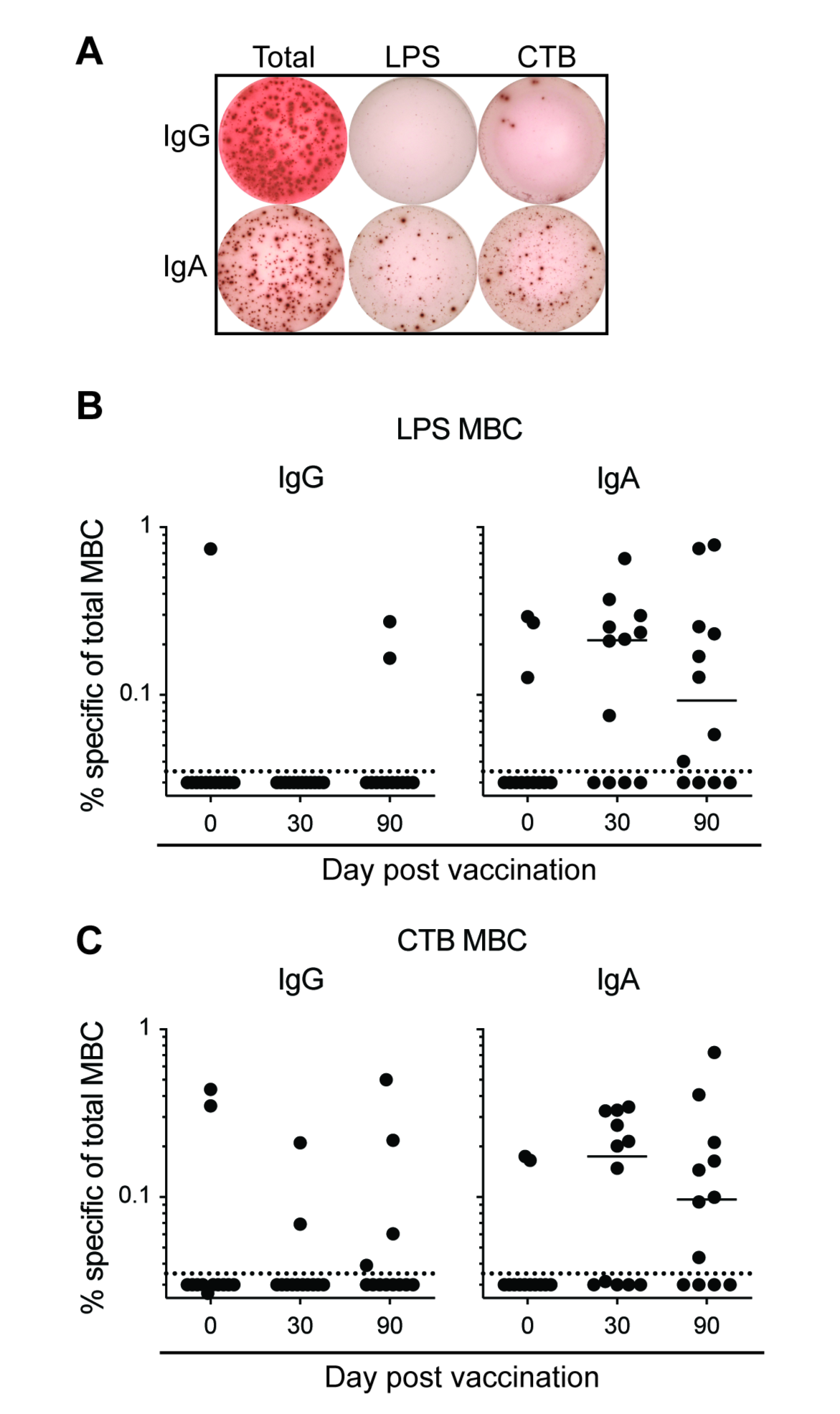

Supplement: S4 Fig — (A) Representative ELISPOT and summary analysis of (B) LPS and (C) CTB specific IgG and IgA memory B cells on days 0, 30, and 90 post vacation for each of the 12 participants. Dashed line indicates limit of detection for the memory B cell assay. (TIF) [file pntd.0009743.s004.tif]

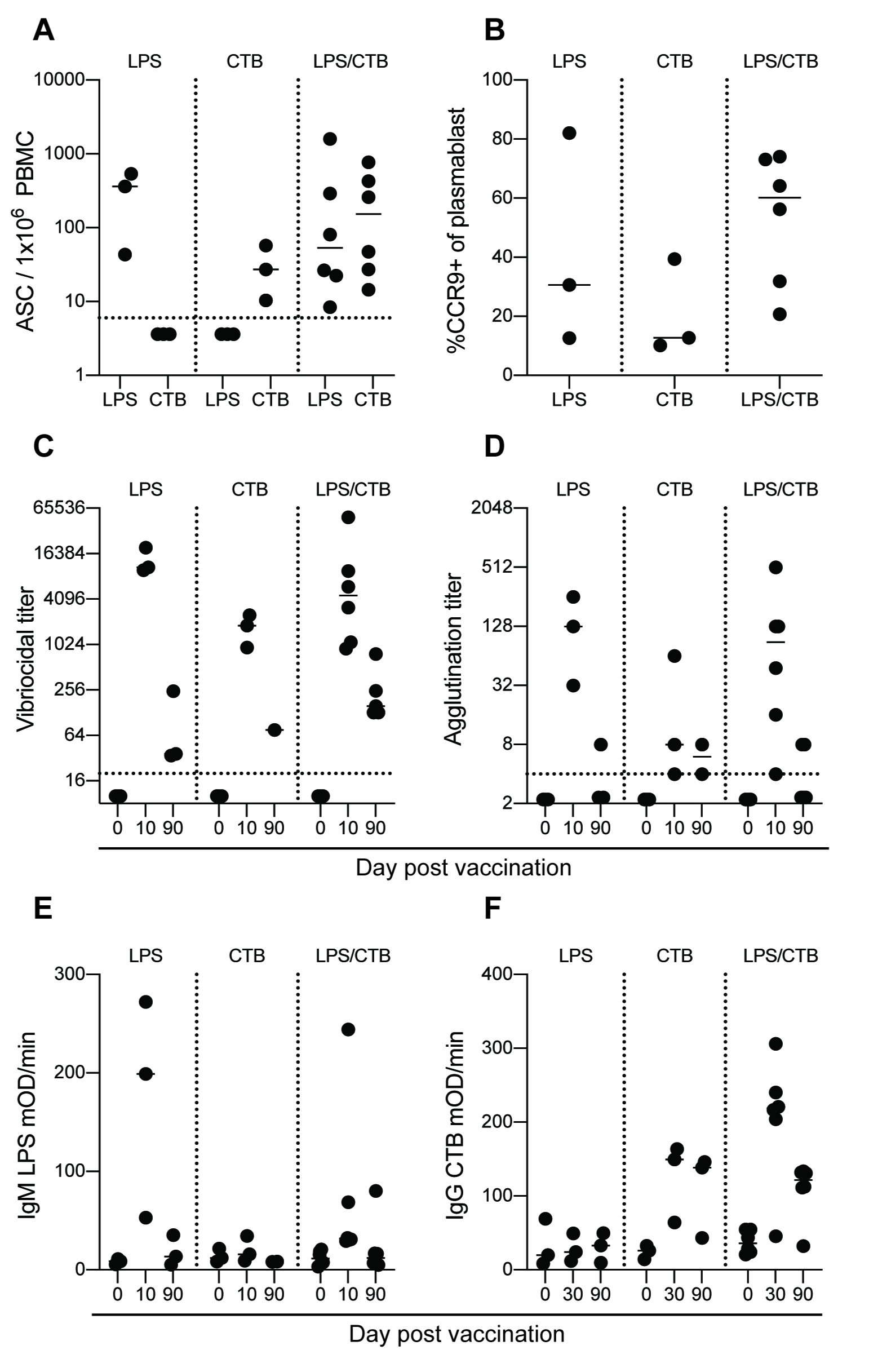

Supplement: S5 Fig — All 12 subjects are divided into LPS only, CTB only, and LPS/CTB double responders based on antibody secreting cell responses to the immunodominant cholera antigens LPS and CTB. (A) Day 7 ASC responses from each subject to LPS and CTB as measured by ELISPOT. (B) Percentage of plasmablasts which were CCR9+ as measured by flow cytometry on 7 days post vaccination for each of the study participants. (C) Vibriocidal titers as measured on day 0, 10, and 90 for each participant. (D) Agglutination titer measured on day 0, 10, and 90 for each participant. (E) IgM anti LPS titers measured on day 0, 10, and 90 for each subject. (F) IgG anti CTB titers measured on day 0, 10, and 90 for each subject. (TIF) [file pntd.0009743.s005.tif]

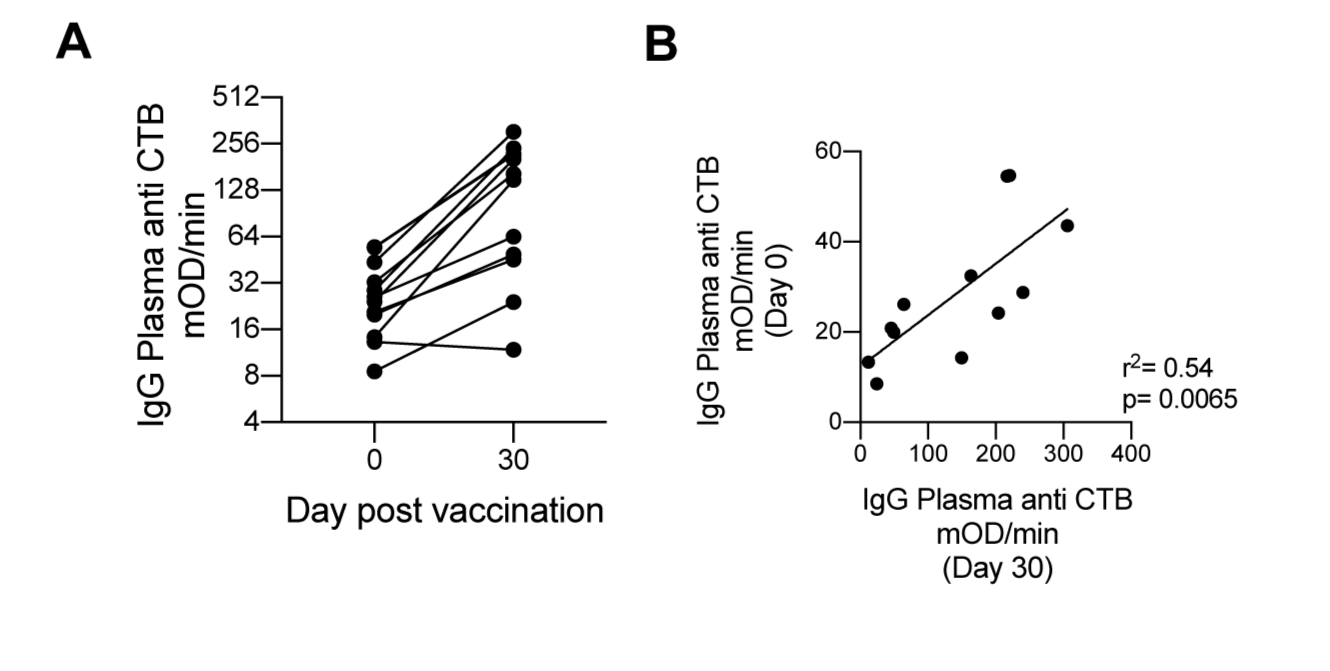

Supplement: S6 Fig — (A) Anti IgG CTB titers as measured by ELISA on day 0 and 30 post vaccination for each subject. (B) Linear regression analysis of IgG anti CTB titers as measured by ELISA on day 0 (x axis) and day 30 (y axis) post vaccination. Significance values are indicated by asterisks (P < 0.05 (*); P < 0.005 (**); P < 0.0005 (***); P <0.0001 (****)). (TIF) [file pntd.0009743.s006.tif]
